# Are spasticity, weakness, selectivity, and passive range of motion related to gait deviations in children with spastic cerebral palsy? A statistical parametric mapping study

**DOI:** 10.1371/journal.pone.0223363

**Published:** 2019-10-11

**Authors:** Eirini Papageorgiou, Cristina Simon-Martinez, Guy Molenaers, Els Ortibus, Anja Van Campenhout, Kaat Desloovere

**Affiliations:** 1 KU Leuven Department of Rehabilitation Sciences, Leuven, Belgium; 2 Clinical Motion Analysis Laboratory, University Hospitals Leuven, Leuven, Belgium; 3 KU Leuven Department of Development and Regeneration, Leuven, Belgium; 4 Department of Orthopedics, University Hospitals Leuven, Leuven, Belgium; University of Bern, SWITZERLAND

## Abstract

This study aimed to identify the relationships between clinical impairments and gait deviations in children with cerebral palsy (CP). A retrospective convenience sample of 367 children with CP was selected (3–18 years old) and divided in two groups based on clinical symptomatology [unilateral (uCP) / bilateral CP (bCP), (n = 167/200)]. All children underwent a three-dimensional gait analysis and a standardized clinical examination. Gait was inspected on a vector level (all sagittal motions combined), and an individual joint level (pelvis, hip, knee and ankle joint motions). Statistical non-parametric mapping was applied to identify specific parts of the gait cycle displaying relationships between the gait deviations of both groups and the impairment scores of spasticity, weakness, selectivity, and passive range of motion. Impairment scores were summarized in two ways: a) composite impairment scores (e.g. combined spasticity of all assessed muscles acting around the hip, knee and ankle joints) and b) joint specific impairment scores (e.g. spasticity of the muscles acting around the knee joint). Results showed that the vector and most of the individual motions were related to the composite scores. Direct and carry-over relationships were found between certain individual motions and joint impairment scores (around the same or neighboring joints, respectively). All correlations were more prominent for children with bCP compared to uCP, especially regarding the relationships of gait deviations with weakness and reduced selectivity. In conclusion, this study enabled the mapping of relationships between clinical impairments and gait deviations in children with CP, by identifying specific parts of the gait cycle that are related to each of these impairments. These results provide a comprehensive description of these relationships, while simultaneously highlighting the differences between the two CP groups. Integration of these findings could lead to a better understanding of the pathophysiology of gait deviations and, eventually, support individualized treatment planning.

## Introduction

Children with spastic cerebral palsy (sCP) suffer primarily from clinical impairments such as increased tone, muscle weakness, diminished selectivity, and joint contractures. These impairments contribute to the abnormal development of functional activities, including gait [1]. Several studies have tried to identify and establish the relationships between the impairments and gait abnormalities, however with quite different methodologies and results [2–14]. The link between impairment and function has been hypothesized as a crucial stepping stone in the identification of an optimal patient-tailored treatment planning [15–17].

Discrepancies in the reported results may stem from the different methodological approaches applied in previous research. Studies have previously focused on exploring the relationships between clinical impairments and various types of gait measures. These measures concerned, on the one hand, distinct gait parameters referring to specific points of the gait cycle, such as minimum or maximum joint angles, or spatiotemporal parameters [3–5,8]. On the other hand, quantified overall measures of gait deviations, such as the gait profile score [10,11,13], gait classification systems [2,12], or entire gait curves [7] have been used for such explorations.

Another possible reason for the discrepancy between the results of previous studies is the considered study samples and their (sometimes) heterogeneous characteristics. In some studies, spasticity scores showed fewer correlations to gait data in comparison to muscle strength or selectivity measurements [3,6], even though there are quite established beliefs in clinical practice as to how spasticity is expected to affect specific parts of sCP gait. For example, diminished dorsiflexion during stance is often linked to spastic plantar flexor muscles; increased knee flexion during stance [17,18] or terminal swing [19] has been related to spastic hamstring muscles. In the past, researchers have evaluated whether clinical measurements correlate with distinct gait parameters in mixed groups of children with either unilateral or bilateral sCP (uCP and bCP, respectively) [3,7], concluding that focusing on distinct groups according to the patients’ clinical symptomatology would potentially be more illuminating [3]. This was further explored by Meyns et al. who found only significant relationships between gait deviations and symptoms of spasticity and muscle weakness in children with bCP [10]. In contrast, Crosbie et al., found that muscle stiffness, strength, spasticity and gait-related spatiotemporal parameters were significantly related to each other in a group of children with uCP [20]. Nieuwenhuys et al. showed the existence of relationships between specific joint gait patterns and impairment measurements in both patient groups [2]. In addition, Holmes et al. highlighted differences between impairment scores, as well as spatiotemporal parameters of the two patient groups [11], further supporting the option to study children with uCP independently from children with bCP.

Variability in results might also be due to the investigated impairment scores or the way these scores have been summarized. Some studies have focused on muscle specific scores (e.g. knee flexors’ spasticity) [3,7,11,12], others on joint specific composite scores (e.g. knee spasticity) [2], while others have created composite scores reflecting the total lower limb’s impairments [6,9,10,21,22]. In general, previous contradicting results support the necessity for additional studies investigating the association between gait deviations and clinical impairments in children with CP.

Apart from the heterogeneity among previously selected parameters, sample characteristics and included impairment scores, the applied statistics might also have been suboptimal to answer such research questions. The focus on specific time-points during the gait cycle, where differences are maximized, could lead to results with uncorrected α levels, resulting in increased false positive findings [23]. Statistical parametric mapping (SPM) is an analysis technique that allows for hypothesis testing across an entire waveform, thus avoiding to commit a “regional focus bias”, and increasing statistical power. Moreover, for exploratory, non-directed hypotheses, covariance among various movement components needs to be properly addressed. To this end, vector field testing has been developed, considering, for example, the motions of all lower limb joints in one plane as components of one vector [24]. SPM has already proven to be sensitive when exploring associations between muscle weakness and the gait of children with Duchenne muscular dystrophy, as well as between movement and clinical impairments in upper limb movement analysis in uCP [25,26].

Given the variability in employed methodologies and outcomes of previous studies and the benefits of SPM, it is relevant to further evaluate to what extent the clinical impairments relate to gait deviations in children with sCP using SPM. This study thereby aimed to explore the correlations between clinical impairments and the entire continuous waveforms of the sagittal plane kinematics of children with either uCP or bCP. The current exploration focused on sagittal plane kinematics, since deviations in this plane are most commonly reported [27–29] and are more repeatable [30–32]. These correlations in question were explored separately for uCP and bCP, since previous studies have provided evidence that impairments in these groups are different, as are the relationships of the impairments with gait deviations [2,10,11]. Specifically, the sagittal plane kinematics of the two patient groups were considered separately and their relationship with lower limb spasticity, muscle weakness, selectivity and contractures (passive range of motion–pROM) were explored by means of SPM analysis, based on two **research questions**:

1. Does the combined sagittal motion of the pelvis, hip, knee, and ankle joint motions (i.e. vector) of each patient group relate to their composite impairment scores (i.e. combination of impairment of all involved muscles)? If yes, does each individual sagittal joint motion also relate to these composite scores?
2. Does each joint motion relate to the respective joint specific impairment scores (‘direct’ relationships; i.e. is knee motion related to knee impairments)? Additionally, does each joint motion also relate to the impairment scores of other joints (‘carry-over’ relationships; i.e. is knee motion related to ankle impairments)?

## Methods

### Participants

A retrospective sample of convenience was selected from the Clinical Motion Analysis Laboratory of the University Hospitals Leuven (Medical Ethical Committee of University Hospitals Leuven—s56036). Under this research project, permission was provided by the Medical Ethical Committee of University Hospitals Leuven to use and further process retrospective patient data that have been acquired during standard medical care, provided that all data would have been a priori anonymized, unless the patients had specifically asked to not be included in any study. The entire sample consisted of 367 children who have had a gait analysis session (n_1_ = 167 children with uCP; n_2_ = 200 children with bCP). All children were diagnosed as spastic CP, were ambulatory (levels I to III on the gross motor function classification system–GMFCS) and had undergone a gait analysis between 3 and 18 years of age. Further inclusion criteria consisted of availability of at least two kinematic trials of good quality (see section Data collection for further details), acquired through three-dimensional gait analysis (3-DGA) and a full dataset available from the clinical examination of the patient on the day of the 3-DGA, including spasticity, muscle weakness, selectivity and pROM of the lower limbs. Exclusion criteria included a botulinum toxin injection treatment session within 6 months preceding the 3-DGA or a lower limb surgery within 2 years before the 3-DGA.

### Data collection

Each patient underwent a standardized 3-DGA, at a self-selected walking speed. The 3-DGA included kinematic, kinetic and EMG analysis during barefoot walking. For the current study, only kinematic data were used. Hereto, ten to fifteen optoelectronic camera’s (Oxford Metrics, Oxford, UK) and two force plates (Advanced Mechanical Technology Inc., USA), embedded in a 10m walkway were used. The reflective markers were placed on the specified anatomical landmarks according to the Plug-In-Gait model [33]. Gait cycles were identified, and kinematics were calculated in Vicon Nexus software (Oxford Metrics, Oxford, UK). The kinematic waveforms were time-normalized, yielding a total of 101 data points for each curve. The quality of the gait trials was checked in a custom-made Matlab^®^ software (The MathWorks, Natick, MA, USA, 2015), where the range of motion (ROM) and the absolute values of the knee varus-valgus angle were evaluated [34]. Trials were, additionally, checked for artifacts or outliers (based on visual inspection of each waveform, outliers were defined in relation to the average graph of each side and the variability around it). Trials were excluded if they displayed a knee varus-valgus ROM ≥ 15^o^, a knee valgus angle ≤ -10^o^ or increased variability in respect to the average graph of each side.

Our sample included two main groups according to topography: uCP and bCP. For children with uCP, only the affected lower limbs were considered, whilst for bCP patients, the most spastic and/or weakest lower limb based on their clinical records was chosen. This decision was taken in order to make the groups comparable, on the assumption that higher impairments would dominate the overall gait pathology. The total sample size resulted in a total of 367 children (one lower limb per child). Based on data availability, 2 or 3 kinematic trials were averaged per patient, from which only sagittal plane kinematics were analyzed.

### Clinical examination

**Fig 1:**
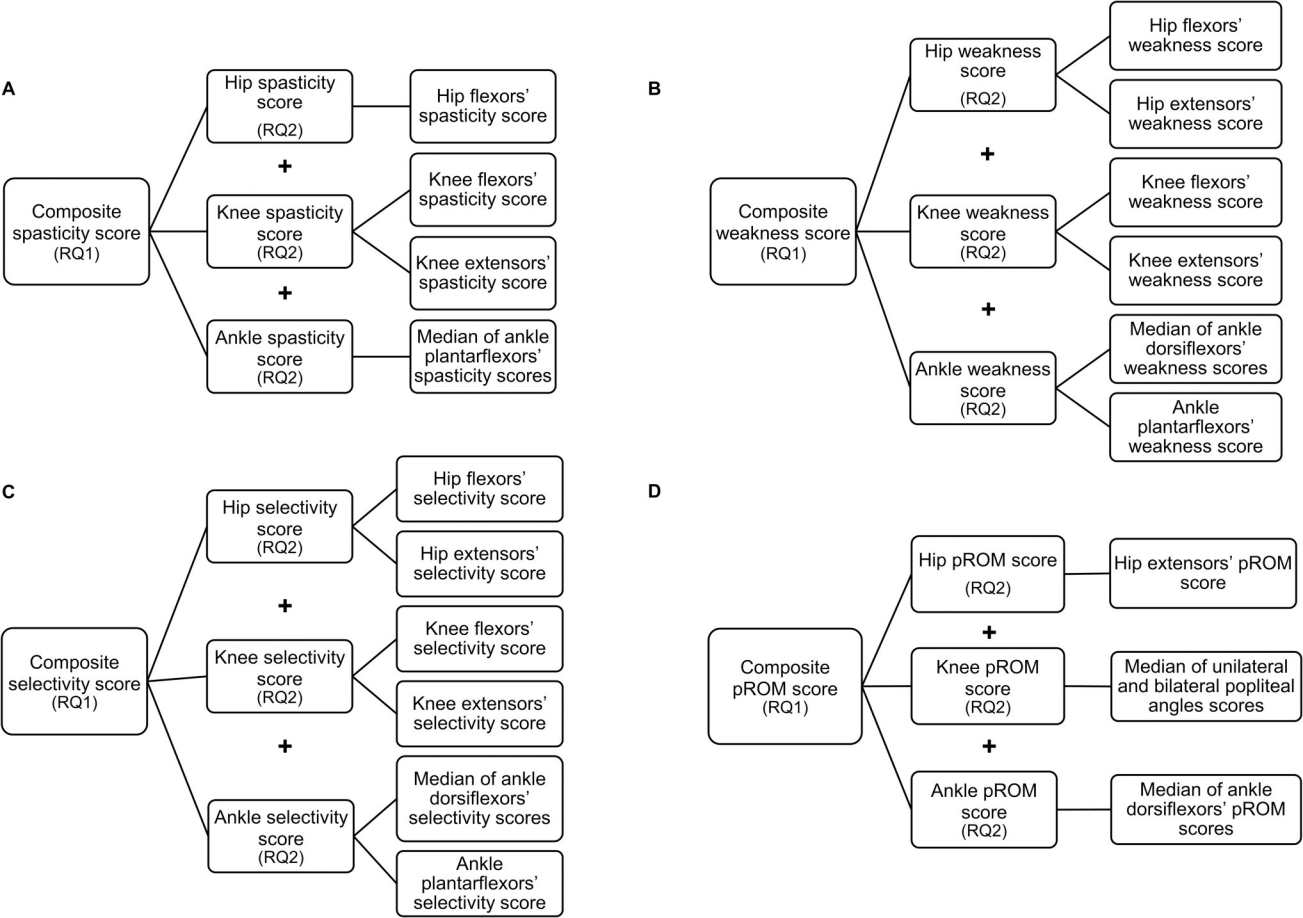
Schematic representation of composite impairment scores, joint specific impairment scores and muscle specific impairment scores. (A) Spasticity, (B) Weakness, (C) Selectivity, (D) pROM.Abbreviations: pROM: passive range of motion; RQ: research question.

At the day of the 3-DGA, all patients underwent a clinical examination focusing mostly on measurements of spasticity, muscle weakness, selectivity, and pROM, performed by experienced physiotherapists. For this study, individual impairment scores were grouped to joint specific impairment scores, and the latter were, subsequently, grouped to total composite scores (from here on indicated as “joint scores” and “composite scores”, respectively–Fig 1) [21]. Thus, per impairment, hip, knee and ankle joint scores, as well as composite scores, were created. In case of synergistic muscles (e.g. gastrocnemius and soleus muscles), a median score was calculated to represent the joint score. When agonists and antagonists act around a joint and both groups are known to be involved in CP (e.g. knee flexors’ and extensors’ weakness), the sum was calculated to represent the joint score. In other cases, the muscle score defined the joint score (e.g. hip extensors’ pROM is the same as the hip pROM score). Individual muscle scores were not considered in this study (apart from cases such as the ‘hip pROM score’). This study aimed primarily at establishing a methodology of exploring such correlations while simultaneously providing a first and summarized overview of relationships between lower limb impairments and gait deviations in children with CP.

**Spasticity** was measured with the Modified Ashworth Scale (MAS) [35], which is a six-point ordinal scale (possible scores: 0, 1, 1.5, 2, 3, 4). The MAS describes the changes in muscle tone experienced by the assessor while applying a passive stretch to the investigated muscles through the full ROM. A score of 0 represents a normal muscle tone, whereas a score of 4 is assigned if no motion is possible due to rigidity. For this study, spasticity scores were grouped according to the joint, resulting in ankle spasticity (median scores of the MAS of the gastrocnemius and soleus muscles–range: 0–4), knee spasticity (sum of the MAS scores of the knee flexors and extensors–range: 0–8), and hip spasticity (only the MAS scores of the hip flexors–range: 0–4). A *composite spasticity score* was defined as the sum of all muscle groups that were evaluated for spasticity (hip, knee and ankle scores, stemming from the hip flexors, knee flexors and extensors and ankle plantar flexors scores, respectively, range: 0–16).

**Muscle weakness** was assessed with the manual muscle testing scale (MMT), which is also a six-point ordinal scale (ranging from 0 to 5; 5 indicating the strongest muscles, able to move against gravity and maximum resistance for the full ROM) [36]. The following MMT scores were taken into consideration: hip weakness (sum of the MMT scores of the hip flexors and extensors–range: 0–10), knee weakness (sum of the MMT scores of the knee flexors and extensors–range: 0–10), and ankle weakness (median MMT scores of dorsiflexors with the knee flexed and extended, summed up with the ankle plantarflexors’ weakness MMT scores–range 0–10). The *composite weakness score* was defined as the sum of the joint weakness scores (range: 0–30).

**Selectivity** was additionally considered as an indication of the ability to move only a specific joint without the involvement of neighboring ones and/or by activating the right muscles [37]. A 5-point ordinal scale defined by Trost et al. was used (from 0 to 2, in increments of 0.5), with a score of 2 describing a perfect selectivity [38]. The same muscles as in the case of MMT scores were taken into consideration comprising the joint scores of hip, knee, and ankle selectivity (range: 0–4 for all joint scores). In addition, a *composite selectivity score* was calculated as the sum of the joint selectivity scores (range: 0–12).

Finally, **pROM** measurements were used to define the potential joint contractures, measured with a goniometer. All goniometric measurements represent the known pathological joint motions (for example, hip extension is typically impaired in children with CP, whereas hip flexion is not). All measurements were, in each of the two groups, transformed to a 3-point ordinal scale ranging from 0 to 2, using their 25^th^ and 75^th^ percentiles as cut-off values (0: values higher than the 75^th^ percentile, indicating no or slight contractures; 1: values between the 25^th^ and 75^th^ percentiles, representing moderate contractures; 2: values below the 25^th^ percentile, used for severe impairments). The Thomas test was applied for the hip pROM (range: 0–2); the median unilateral and bilateral popliteal angle were measured for the knee pROM (range: 0–2), and the ankle pROM was represented by the median of the ankle dorsiflexion pROM with the knee both flexed and extended (range: 0–2) [38]. Subsequently, *a composite pROM score* was also calculated as the sum of the joint pROM scores (range: 0–6).

### Statistical analysis

In order to explore the demographic and clinical data of the two patient cohorts, descriptive statistics were used. Additionally, the normality distribution of age was tested with the Shapiro-Wilk test, the differences between groups were tested with the Mann-Whitney U test, the differences between frequency distributions with Chi-square test (χ^2^) and correlations among the impairment scores with Spearman’s rank correlations (IBM SPSS Statistics for Windows, version 24—IBM Corp., Armonk, N.Y., USA). For the correlation between sagittal plane kinematics and impairment scores, SPM was used (SPM1d version 0.4, available for download at http://www.spm1d.org/) in Matlab^®^ (The MathWorks, Natick, MA, USA, 2015). Random field theory was used to calculate the critical threshold that the test statistic would cross in alpha % of experiments (α = 0.05) involving temporally smooth, random data [23,39]. When the critical threshold was crossed, suprathreshold clusters were formed. In that case, important information concerning the p-value, the extent (percentage of the gait cycle) and the location (first and last points of the cluster) was extracted and reported. The **first research question** investigated whether a vector component (consisting of the combined sagittal motion of the pelvis, hip, knee and ankle joint motions) of each group (uCP and bCP) relates to their *composite scores*. If the vector was associated with the impairments, subsequent post-hoc analyses of each joint motion were computed [40]. In order to identify the relationships between sagittal plane motion in both uCP and bCP and each of the *composite scores*, the non-parametric Canonical Correlation analysis (SnPM{X2}) was used (α = 0.05), which is analog to linear regression. Thereafter, if significant correlations were identified, post-hoc scalar field non-parametric linear regressions for each vector component (i.e. pelvis, hip, knee, ankle) (SnPM{t}) were computed at a corrected alpha threshold (α = 0.05/4 = 0.0125).

The **second research question** explored whether each joint motion of each patient group is associated to the joint scores of the respective joint or the scores of the neighboring joints. To achieve this, scalar field non-parametric linear regressions were performed (SnPM{t}) (α = 0.05 or 0.025 depending on whether one or two muscle groups comprised each impairment score). For example, when investigating the relationship between hip motion and hip spasticity, only the hip flexors were taken into consideration (with α = 0.05). On the contrary, for the relationship between hip motion and knee spasticity, two muscle groups (knee flexors and extensors) were individually explored with a non-parametric linear regression (with α = 0.025).

Based on a first qualitative inspection of the results and the opinions of the involved clinical experts, it was decided that only clusters of at least 3% of the total gait cycle were considered as displaying a clinical significance on top of the statistical one. A series of example data showed that clusters <3% were quite unstable (diminishing or disappearing in the different sub-samples). All impairment scores originate from ordinal scales, hence the non-parametric version of SPM (i.e. statistical non-parametric mapping–SnPM; with 10000 iterations) was applied.

**Fig 2:**
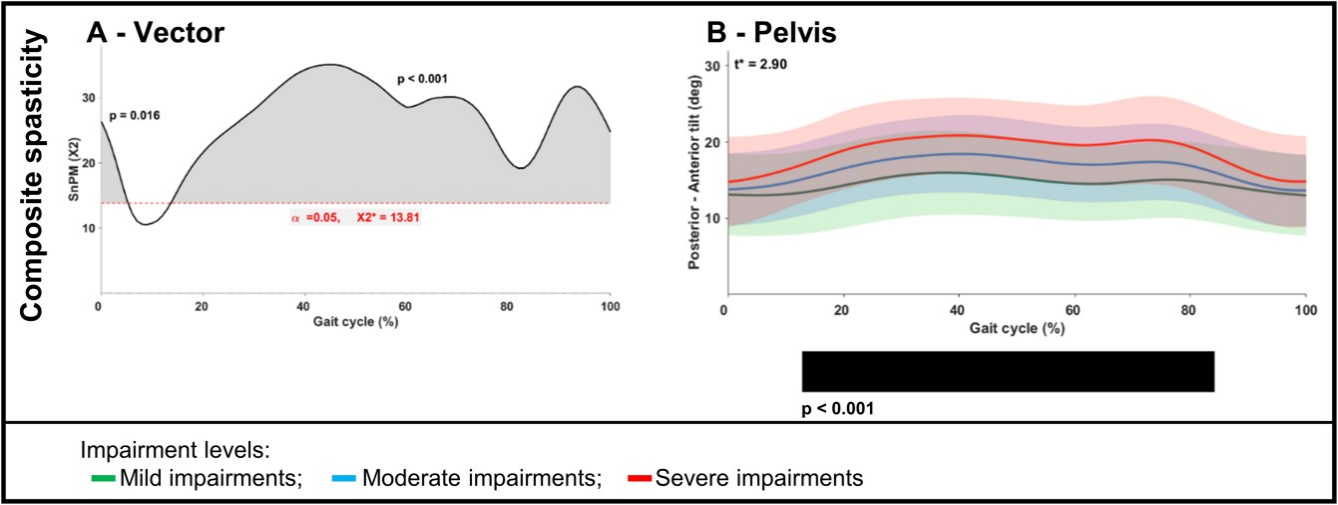
Statistical non-parametric mapping output example for the correlation of the composite spasticity score and gait in children with uCP. **(A)** Non-parametric Canonical Correlation analysis (SnPM{X2}) output example for the correlation of sagittal plane kinematics (vector with four components) with the composite spasticity score in children with uCP. Two suprathreshold clusters were found (cluster 1: 5%, p = 0.016; cluster 2: 86%, p < 0.001). The bold black line represents the computed X2-curve, the dashed red line indicates the random field theory threshold calculated for this test (at 13.81 for α < 0.05). The composite spasticity score associated with the sagittal motion vector of children with uCP for a total of 91%. **(B)** Post-hoc scalar field non-parametric linear regression analysis (SnPM{t}) output example for the correlation between pelvic motion and the composite spasticity score in children with uCP (α = 0.0125). One cluster was identified (71%, p < 0.001). The impairments were divided into 3 categories, depicted as mean (bold line) and standard deviation (translucent area): mild (green color), moderate (blue) and severe (red), indicating that the higher the impairment, the stronger the. The black bars underneath the figures represent the suprathreshold clusters that were formed when the critical threshold (t*) was exceeded and the null hypothesis was, therefore, rejected. Abbreviation: uCP: unilateral cerebral palsy.

An example of these illustrations is depicted in Fig 2, representing the relationship between the *composite spasticity score* and the vector component, as well as the post-hoc linear regression analysis between the *composite spasticity score* and the motion of the pelvis. The SnPM output will be presented in this manner throughout the manuscript.

## Results

### Patient characteristics

The detailed patient characteristics can be found in Table 1. Children with both unilateral and bilateral CP were included in this study, but were analyzed separately based on the previously suggested differences in relationships between the impairments and the gait deviations of these two groups. This was further supported by the fact that the two groups were similar regarding age and gender, but differed clearly, however, with regard to GMFCS levels, treatment history and the majority of their impairment scores. The only scores that were similar between the two groups were the ankle weakness and selectivity scores, and all the pROM impairment scores (apart from the hip pROM). The majority of the impairment scores showed significant correlations among them (Tables A-C in S2 File).

**Table 1:** Participants' characteristics (N = 367) and between groups comparisons for all characteristics.

		uCP (n = 167)	bCP (n = 200)	p (MWU)	p (χ^2^)
**Age [years]**	Median (IQR)	9,31 (6,52–12,21)	9,04 (6,79–11,91)	0.883	
**Gender**	Boys [n (%)]	98 (59%)	130 (65%)		0.214
	Girls [n (%)]	69 (41%)	70 (35%)		
**GMFCS**	I [n (%)]	134 (80%)	86 (43%)		<0.001*
	II [n (%)]	33 (20%)	76 (38%)		
	III [n (%)]	-	38 (19%)		
**Analyzed limb**	Left [n (%)]	81 (49%)	92 (46%)		
**Kinematic trials used**	3 trials [n (%)]	142 (85%)	175 (88%)		
	2 trial [n (%)]	25 (15%)	25 (12%)		
**Previous treatments**	BTX (> 6 months before 3-DGA) [n (%)]	84 (50%)	147 (74%)		<0.001*
** **	Lower limb surgery (> 2 years before 3-DGA) [n (%)]	8 (5%)	37 (19%)		<0.001*
**Spasticity (range)**					
Composite spasticity^a^ (0–16)	Median (IQR)	3.5 (3–4.5)	6 (4.5–7)	<0.001*	
Hip spasticity (0–4)		0 (0–1)	1 (1–1.5)	<0.001*	
Knee spasticity (0–8)		1.5 (1–2)	3 (2–3.5)	<0.001*	
Ankle spasticity (0–4)		1.5 (1.5–2)	2 (1.5–2)	<0.001*	
**Weakness (range)**					
Composite weakness^b^ (0–30)	Median (IQR)	22 (20–24)	21 (18–23)	<0.001*	
Hip weakness (0–10)		8 (7–8)	7 (6–8)	<0.001*	
Knee weakness (0–10)		8 (7–8)	7 (6–8)	<0.001*	
Ankle weakness (0–10)		6 (5–7)	6 (5–8)	0.775	
**Selectivity (range)**					
Composite selectivity^**c**^ (0–12)	Median (IQR)	10.5 (9–11)	9.5 (7.5–11)	0.001*	
Hip selectivity (0–4)		4 (3.5–4)	3.5 (3–4)	<0.001*	
Knee selectivity (0–4)		4 (3.5–4)	3.5 (2.5–4)	<0.001*	
Ankle selectivity (0–4)		2.5 (2–3)	2.5 (2–3.5)	0.940	
**pROM (range)**					
Composite pROM^**d**^ (0–6)	Median (IQR)	3 (2–4)	3 (2–4)	0.145	
Hip pROM (0–2)		0 (0)	0 (0–1.5)	<0.001*	
Knee pROM (0–2)		1 (1–2)	1 (1–2)	0.172	
Ankle pROM (0–2)		1 (1–2)	1 (1–2)	0.082	

*α = 0.001

uCP: unilateral cerebral palsy; bCP: bilateral cerebral palsy; MWU: Mann-Whitney U test; χ^2^: Pearson’s chi squared; IQR: interquartile range; GMFCS: gross motor function classification system; BTX: botulinum toxin type A treatments; 3-DGA: three-dimensional gait analysis; pROM: passive range of motion.

^a^ Composite spasticity concerned the hip flexors, the knee flexors and extensors and the ankle plantarflexors

^b, c^ Composite weakness and selectivity concerned the hip flexors and extensors, the knee flexors and extensors and the ankle plantar and dorsiflexors

^d^ Composite pROM concerned the hip extensors, the knee popliteal angle and the ankle dorsiflexors.

### Relation of the composite impairment scores with the combined sagittal motion of the lower limb joints and with each individual joint motion (research question 1)

**Fig 3:**
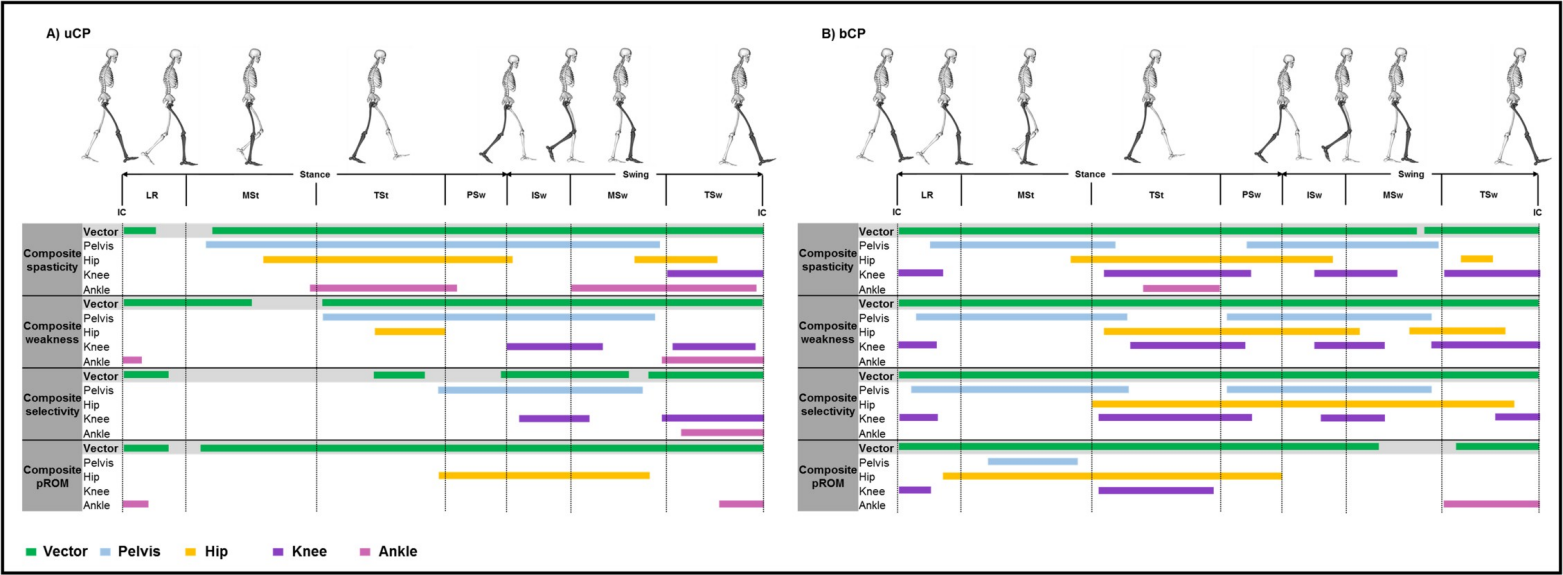
**Summary scheme of all relationships of composite impairment scores with sagittal plane motion in children with A) uCP and b) bCP.** The green lines represent the suprathreshold clusters that were identified during an entire gait cycle when associating with the sagittal plane motion (i.e. vector with four components–Canonical Correlation analysis, α = 0.05). The blue, yellow, purple and pink lines represent the correlations with the sagittal plane motions of the pelvis, hip, knee and ankle joints, respectively (post-hoc scalar field non-parametric linear regression analyses, α = 0.0125) with the composite impairment scores of spasticity, weakness, selectivity and pROM. Abbreviations: uCP: unilateral cerebral palsy; bCP: bilateral cerebral palsy; IC: initial contact; LR: loading response; MSt: midstance; TSt: terminal stance; PSw: preswing; ISw: initial swing; MSw: midswing; TSw: terminal swing; pROM: passive range of motion.

Correlations between the *composite scores* and the vector component of sagittal plane kinematics were found for all performed analyses (Tables 2 and 3, Fig 3, Figs A and B in S1 File). These results indicated that higher *composite scores of spasticity*, *weakness*, *selectivity and pROM* correlated to increased sagittal plane deviations in both patient groups.

**Table 2:** Composite scores and their relationship with the vector component and the individual movements of the pelvis, hip, knee and ankle in the sagittal plane in children with unilateral cerebral palsy.

	Vector^a^		Pelvis^b^ (Posterior—Anterior tilt)		Hip^b^ (Extension—Flexion)		Knee^b^ (Extension—Flexion)		Ankle^b^ (Plantar flexion—Dorsiflexion)	
	n clusters (X2*)	% curve (range)	n clusters (t*)	% curve (range)	n clusters (t*)	% curve (range)	n clusters (t*)	% curve (range)	n clusters (t*)	% curve (range)
Composite spasticity	2 (13.81)	5% (0–5%)	1 (2.90)	71% (13–84%)	2 (3.09)	39% (22–61%)	1 (3.27)	15% (85–100%)	2 (3.12)	23% (29–52%)
		86% (14–100%)				13% (80–93%)				29% (70–99%)
Composite weakness	2 (13.62)	20% (0–20%)	1 (2.97)	52% (31–83%)	1 (3.13)	11% (39–50%)	2 (3.29)	15% (60–75%)	2 (3.11)	3% (0–3%)
		69% (31–100%)						13% (86–99%)		16% (84–100%)
Composite selectivity	4 (13.63)	7% (0–7%)	1 (2.94)	32% (49–81%)	-		2 (3.33)	11% (62–73%)	1 (3.13)	13% (87–100%)
		8% (39–47%)						16% (84–100%)		
		20% (59–79%)								
		18% (82–100%)								
Composite pROM	2 (13.62)	7% (0–7%)	-		1 (3.15)	33% (49–82%)	-		2 (3.21)	4% (0–4%)
		88% (12–100%)								7% (93–100%)

pROM: passive range of motion.

^a^ α <0.05

^b^ α < 0.0125

p ≤ 0.05 (orange)

p ≤ 0.01 (blue)

p ≤ 0.001 (green)

Vector: vector component consisting of the combination of the individual movements of the pelvis, the hip, the knee and the ankle joints in the sagittal plane; n clusters: number of identified suprathreshold clusters; X2*/t*: critical thresholds needed to reject the null hypothesis; % curve (range): extent of the identified suprathreshold cluster (start and end points of the identified cluster); Composite spasticity: sum of spasticity scores of the hip flexors, the knee flexors and extensors and the ankle plantarflexors; Composite weakness: sum of weakness scores of the hip flexors and extensors, the knee flexors and extensors and the ankle plantar and dorsiflexors; Composite selectivity: sum of selectivity scores of the hip flexors and extensors, the knee flexors and extensors and the ankle plantar and dorsiflexors; Composite pROM: sum of pROM scores of the hip extensors, the knee popliteal angle and the ankle dorsiflexors.

**Table 3:** Composite scores and their relationship with the vector component and the individual movements of the pelvis, hip, knee and ankle in the sagittal plane in children with bilateral cerebral palsy.

	Vector^a^		Pelvis^b^ (Posterior—Anterior tilt)		Hip^b^ (Extension—Flexion)		Knee^b^ (Extension—Flexion)		Ankle^b^ (Plantar flexion—Dorsiflexion)	
	n clusters (X2*)	% curve (range)	n clusters (t*)	% curve (range)	n clusters (t*)	% curve (range)	n clusters (t*)	% curve (range)	n clusters (t*)	% curve (range)
Composite spasticity	2 (13.50)	81% (0–81%)	2 (3.02)	29% (5–34%)	2 (3.12)	41% (27–68%)	4 (3.27)	7% (0–7%)	1 (3.10)	12% (38–50%)
		18% (82–100%)		30% (54–84%)		5% (88–93%)		23% (32–55%)		
								13% (65–78%)		
								15% (85–100%)		
Composite weakness	1 (13.55)	100% (0–100%)	2 (3.01)	33% (3–36%)	2 (3.16)	40% (32–72%)	4 (3.24)	6% (0–6%)	-	
				32% (51–83%)		15% (80–95%)		18% (36–54%)		
								11% (65–76%)		
								17% (83–100%)		
Composite selectivity	1 (13.49)	100% (0–100%)	2 (2.93)	34% (2–36%)	1 (3.14)	66% (30–96%)	4 (3.15)	6% (0–6%)	-	
				32% (51–83%)				24% (31–55%)		
								10% (66–76%)		
								17% (83–100%)		
Composite pROM	2 (13.59)	75% (0–75%)	1 (2.94)	14% (14–28%)	1 (3.13)	53% (7–60%)	2 (3.20)	5% (0–5%)	1 (3.13)	15% (85–100%)
		13% (87–100%)						18% (31–49%)		

pROM: passive range of motion.

^a^ α <0.05

^b^ α < 0.0125

p ≤ 0.01 (blue)

p ≤ 0.001 (green)

Vector: vector component consisting of the combination of the individual movements of the pelvis, the hip, the knee and the ankle joints in the sagittal plane; n clusters: number of identified suprathreshold clusters; X2*/t*: critical thresholds needed to reject the null hypothesis; % curve (range): extent of the identified suprathreshold cluster (start and end points of the identified cluster); Composite spasticity: sum of spasticity scores of the hip flexors, the knee flexors and extensors and the ankle plantarflexors; Composite weakness: sum of weakness scores of the hip flexors and extensors, the knee flexors and extensors and the ankle plantar and dorsiflexors; Composite selectivity: sum of selectivity scores of the hip flexors and extensors, the knee flexors and extensors and the ankle plantar and dorsiflexors; Composite pROM: sum of pROM scores of the hip extensors, the knee popliteal angle and the ankle dorsiflexors.

In **uCP**, the vector component correlation with the *composite spasticity score* resulted in two suprathreshold clusters (extent 5%—p = 0.016 and 86%—p < 0.001, respectively) (Table 2, Fig 3, and Fig A in S1 File). Post-hoc analysis revealed that the *composite spasticity score* associated to an increased *pelvic* anterior tilt during most of the gait cycle (71%—p < 0.001), increased *hip* flexion (39%—p < 0.001; 13%—p = 0.003), increased *knee* flexion (terminal swing: 15%—p < 0.001) and reduced *ankle* dorsiflexion (23%—p < 0.001; 29%—p < 0.001). Correlations between the vector component and the *composite weakness score* were also found for the majority of the gait cycle (20%—p = 0.002; 69%—p < 0.001). Post-hoc analysis indicated a relation between higher *composite weakness score* and increased *pelvic* anterior tilt (52%—p < 0.001), increased *hip* flexion (hip: 11% in terminal stance–p = 0.003) and impaired *knee* flexion (decreased knee flexion: 15% in initial swing—p < 0.001; increased knee flexion: 13% in terminal swing—p < 0.001), and reduced *ankle* dorsiflexion (3%—p = 0.003; 16%—p < 0.001). The *composite selectivity and pROM scores* were related to the sagittal motion vector component (*composite selectivity score*: 7%—p = 0.005; 8%—p = 0.034; 20%—p < 0.001; 18%—p < 0.001; *composite pROM score*: 7%—p = 0.015; 88%—p < 0.001). Reduced selectivity was related to increased *pelvic* anterior tilt (32%—p < 0.001), increased *knee* flexion (11%—p = 0.001 and 16%—p <0.001), and reduced *ankle* dorsiflexion (13%—p < 0.001). The *composite pROM score* associated with increased *hip* flexion (33%—p < 0.001) and moderately impaired selectivity during *ankle* dorsiflexion (4%—p = 0.003; 7%—p = 0.004). Each joint motion correlated with only one *composite impairment score* during specific parts of the gait cycle [*composite spasticity score*: increased *pelvic* anterior tilt in midstance (13–30%), increased *hip* flexion in mid- and terminal stance (22–38%) and terminal swing (83–93%), reduced *ankle* dorsiflexion in terminal stance (29–52%) and midswing (70–83%); *composite pROM score*: increased *hip* flexion in initial and midswing (62–79%)].

In **bCP**, the vector component associated with all *composite scores* (*spasticity*: 81%—p < 0.001 and 18%—p < 0.001; *weakness*: 100%—p < 0.001; *selectivity*: 100%—p < 0.001; *pROM*: 75% = p < 0.001 and 13%—p = 0.004) (Table 3, Fig 3, Fig B in S1 File). In general, all *composite scores* of the bCP children showed a relationship with increased *pelvic* anterior tilt, increased *hip* flexion and increased *knee* flexion at initial contact and during loading response, from mid to late stance and during terminal swing and reduced *knee* flexion during initial and midswing. Reduced *ankle* dorsiflexion was further associated with the *composite spasticity score* (12%—p = 0.002) and the *composite pROM score* (15%—p < 0.001). No correlations between the composite weakness and selectivity scores and *ankle* motion were found in the bCP group. The c*omposite spasticity score* was the only score that correlated with reduced *ankle* dorsiflexion during terminal stance (38–50%). *Composite pROM score* was uniquely correlated with increased *hip* flexion from loading response to midstance (7–26%) and with reduced *ankle* dorsiflexion during terminal swing (85–100%).

### Relation of joint motions to their respective joint specific impairment scores and to the impairment scores of other joints (research question 2)

**Fig 4:**
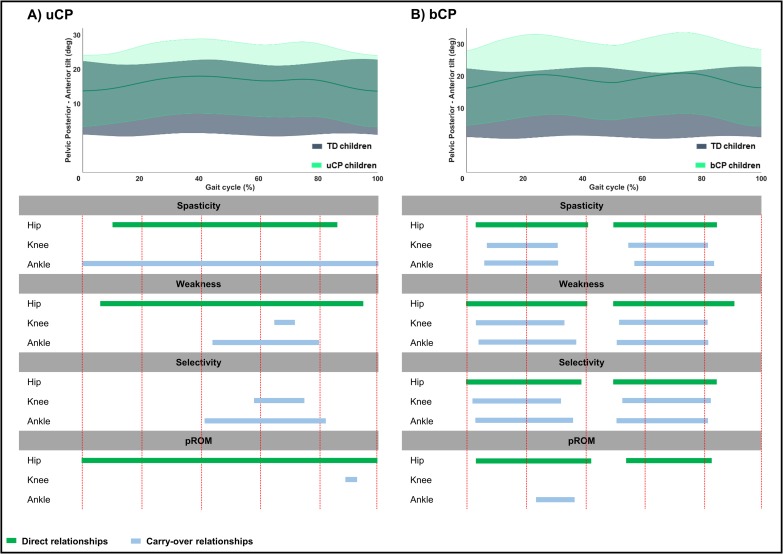
**Summary scheme of all relationships between joint specific impairment scores and pelvic sagittal motion in children with A) uCP and B) bCP.** The grey and light green areas represent the mean kinematic angles of TD and uCP or bCP children, respectively. The green bars show the direct relationships between the pelvic sagittal motion and the hip joint impairment scores; the blue bars depict the carry-over relationships between the pelvic sagittal motion and the knee or ankle joint impairment scores across an entire gait cycle. Abbreviations: uCP: unilateral cerebral palsy; bCP: bilateral cerebral palsy; TD: typically developing children; pROM: passive range of motion.

**Fig 5:**
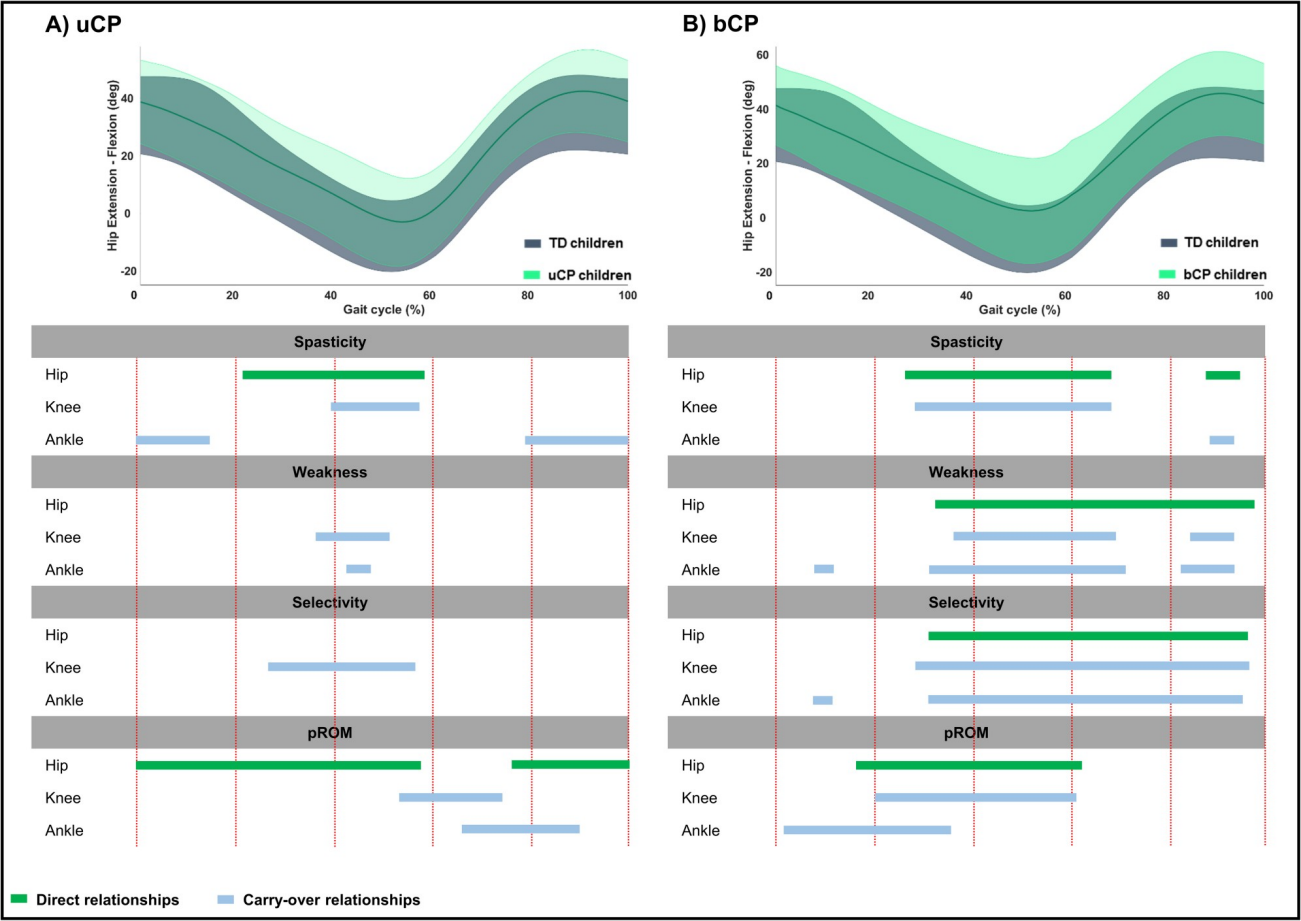
**Summary scheme of all relationships between joint specific impairment scores and hip sagittal motion in children with A) uCP and B) bCP.** The grey and light green areas represent the mean kinematic angles of TD and uCP or bCP children, respectively. The green bars show the direct relationships between the hip sagittal motion and the hip joint impairment scores; the blue bars depict the carry-over relationships between the hip sagittal motion and the knee or ankle joint impairment scores across an entire gait cycle. Abbreviations: uCP: unilateral cerebral palsy; bCP: bilateral cerebral palsy; TD: typically developing children; pROM: passive range of motion.

**Fig 6:**
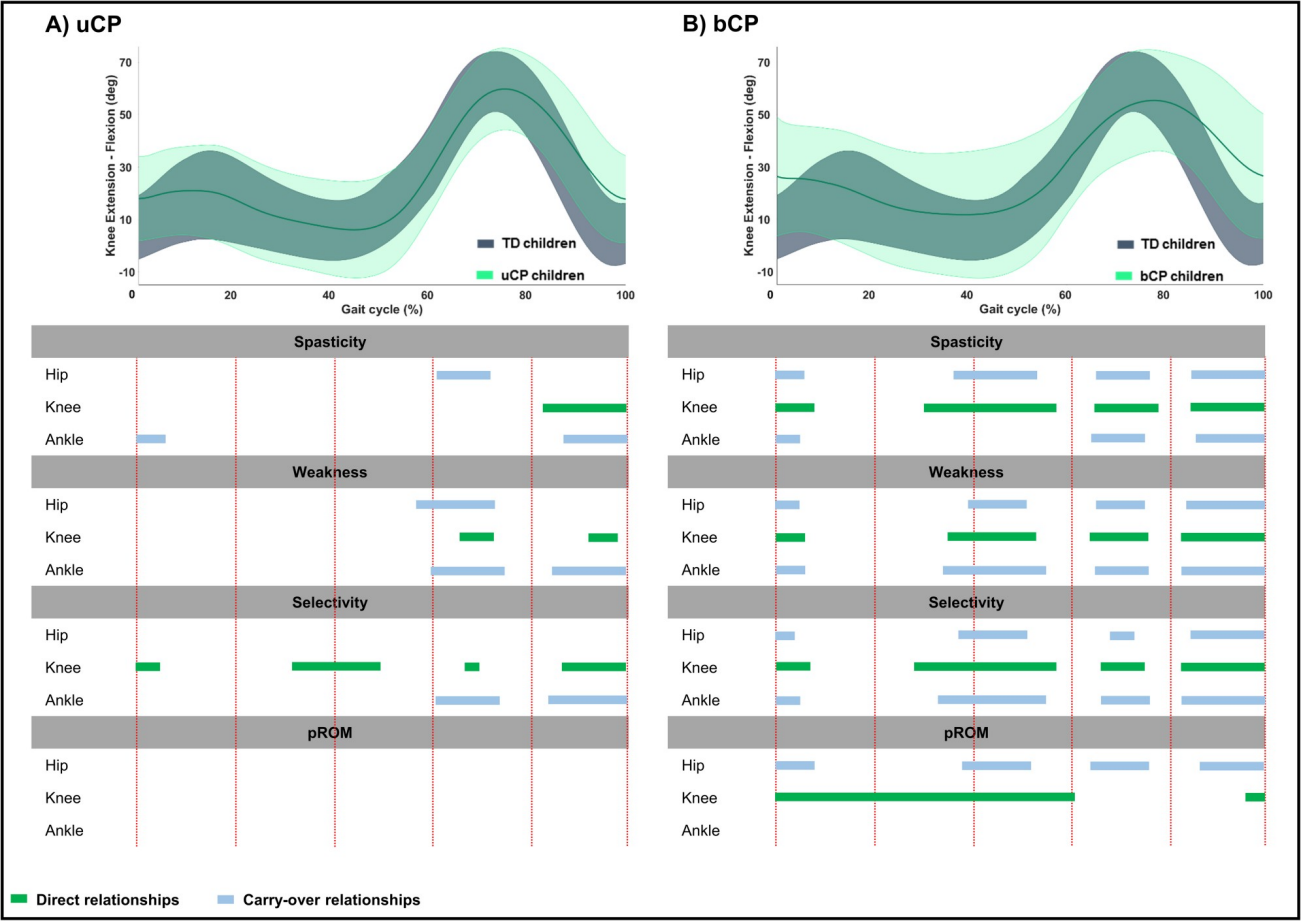
**Summary scheme of all relationships between joint specific impairment scores and knee sagittal motion in children with A) uCP and B) bCP.** The grey and light green areas represent the mean kinematic angles of TD and uCP or bCP children, respectively. The green bars show the direct relationships between the knee sagittal motion and the knee joint impairment scores; the blue bars depict the carry-over relationships between the knee sagittal motion and the hip or ankle joint impairment scores across an entire gait cycle. Abbreviations: uCP: unilateral cerebral palsy; bCP: bilateral cerebral palsy; TD: typically developing children; pROM: passive range of motion.

**Fig 7:**
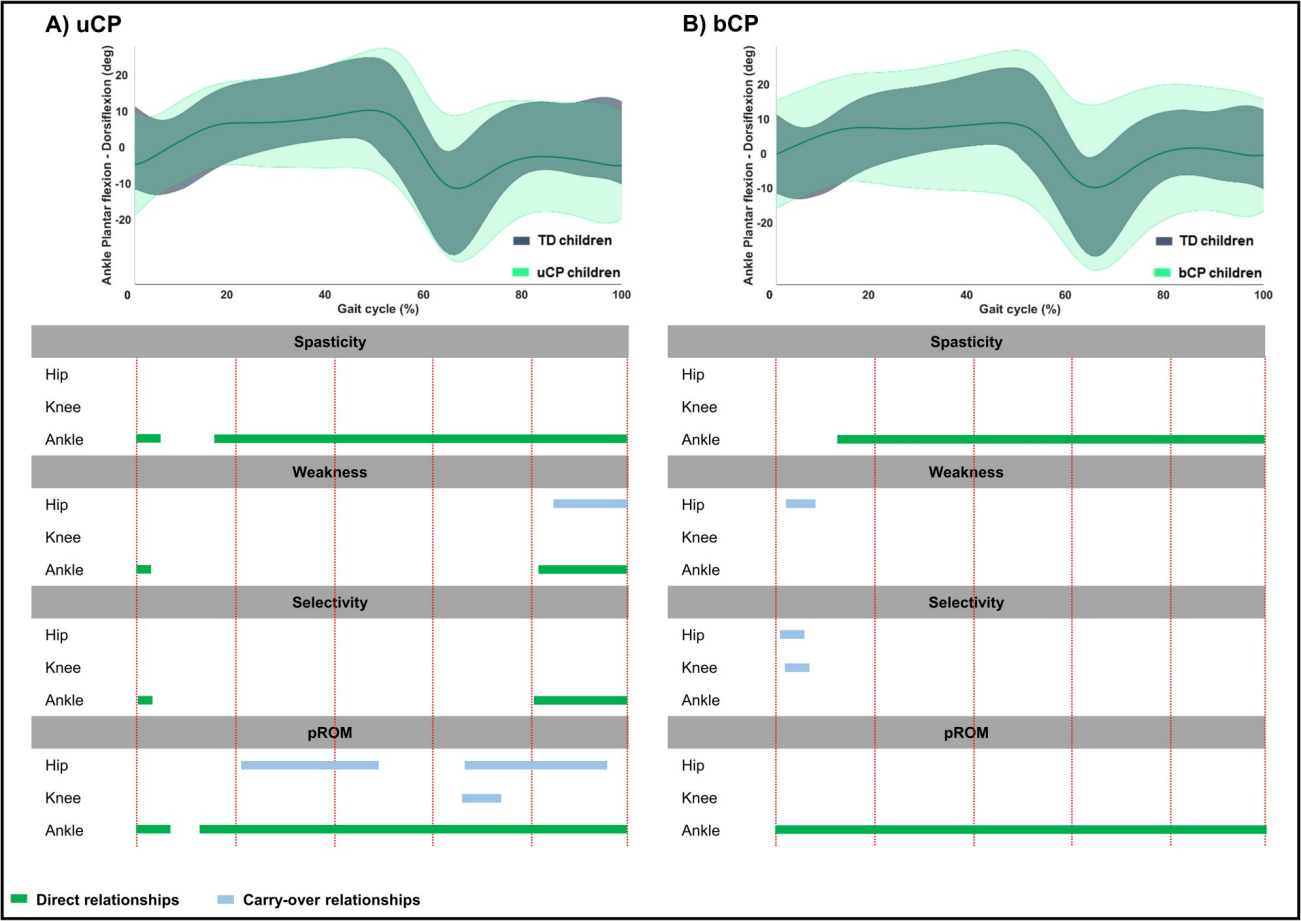
**Summary scheme of all relationships between joint specific impairment scores and ankle sagittal motion in children with A) uCP and B) bCP.** The grey and light green areas represent the mean kinematic angles of TD and uCP or bCP children, respectively. The green bars show thedirect relationships between the ankle sagittal motion and the ankle joint impairment scores; the blue bars depict the carry-over relationships between the ankle sagittal motion and the hip or knee joint impairment scores across an entire gait cycle. Abbreviations: uCP: unilateral cerebral palsy; bCP: bilateral cerebral palsy; TD: typically developing children; pROM: passive range of motion.

Correlations between each joint motion and the joint scores were also found for the majority of the analyses (Tables 4 and 5, Figs 4–7, Figs C and D in S1 File).

**Table 4:** Joint impairment scores and their relationship with the individual movements of the pelvis, hip, knee and ankle in the sagittal plane in children with unilateral cerebral palsy.

		Pelvis (Posterior—Anterior tilt)		Hip (Extension—Flexion)		Knee (Extension—Flexion)		Ankle (Plantarflexion—Dorsiflexion)	
		n clusters (t*)	% curve (range)	n clusters (t*)	% curve (range)	n clusters (t*)	% curve (range)	n clusters (t*)	% curve (range)
Spasticity	Hip^a^	1 (2.43)	76% (10–86%)	1 (2.64)	37% (22–59%)	1 (2.73)	11% (61–72%)	-	
	Knee^b^	-		1 (2.82)	18% (40–58%)	1 (3.05)	17% (83–100%)	-	
	Ankle^a^	1 (3.24)	100% (0–100%)	2 (2.59)	15% (0–15%)	2 (2.77)	6% (0–6%)	2 (2.60)	5% (0–5%)
					21% (79–100%)		13% (87–100%)		84% (16–100%)
Weakness	Hip^b^	1 (2.71)	89% (6–95%)	-		1 (3.03)	16% (57–73%)	1 (2.87)	15% (85–100%)
	Knee^b^	1 (2.71)	7% (65–72%)	1 (2.90)	15% (37–52%)	2 (3.03)	7% (66–73%)	-	
							6% (92–98%)		
	Ankle^b^	1 (2.63)	36% (44–80%)	1 (2.89)	5% (43–48%)	2 (3.01)	15% (60–75%)	2 (2.90)	3% (0–3%)
							15% (85–100%)		18% (82–100%)
Selectivity	Hip^b^	-		-		-		-	
	Knee^b^	1 (2.72)	17% (58–75%)	1 (2.91)	30% (27–57%)	4 (3.09)	5% (0–5%)	-	
							18% (32–50%)		
							3% (67–70%)		
							13% (87–100%)		
	Ankle^b^	1 (2.65)	41% (41–82%)	-		2 (3.06)	13% (61–74%)	2 (2.96)	3% (0–3%)
							16% (84–100%)		19% (81–100%)
pROM	Hip^a^	1 (3.03)	100% (0–100%)	2 (2.60)	58% (0–58%)	-		2 (2.59)	28% (21–49%)
					24% (76–100%)				29% 67–96%)
	Knee^a^	1 (2.40)	4% (89–93%)	1 (2.63)	21% (53–74%)	-		1 (2.61)	8% (66–74%)
	Ankle^a^	-		1 (2.62)	24% 66–90%)	-		2 (2.70)	7% (0–7%)
									87% (13–100%)

pROM: passive range of motion.

^a^ α <0.05

^b^ α < 0.0125

p ≤ 0.05 (orange)

p ≤ 0.01 (blue)

p ≤ 0.001 (green)

n clusters: number of identified suprathreshold clusters; t*: critical threshold needed to reject the null hypothesis; % curve (range): extent of the identified suprathreshold cluster (start and end points of the identified cluster); Hip spasticity: spasticity of the hip flexors; Knee spasticity: spasticity of the knee flexors and extensors; Ankle spasticity: spasticity of the ankle plantarflexors; Hip weakness: weakness of the hip flexors and extensors; Knee weakness: weakness of the knee flexors and extensors; Ankle weakness: weakness of the ankle plantar and dorsiflexors; Hip selectivity: selectivity of the hip flexors and extensors; Knee selectivity: selectivity of the knee flexors and extensors; Ankle selectivity: selectivity of the ankle plantar and dorsiflexors; Hip pROM: pROM of the hip extensors, Knee pROM: knee popliteal angle; Ankle pROM: pROM of the ankle dorsiflexors.

**Table 5:** Joint impairment scores and their relationship with the individual movements of the pelvis, hip, knee and ankle in the sagittal plane in children with bilateral cerebral palsy.

		Pelvis (Posterior—Anterior tilt)		Hip (Extension—Flexion)		Knee (Extension—Flexion)		Ankle (Plantarflexion—Dorsiflexion)	
		n clusters (t*)	% curve (range)	n clusters (t*)	% curve (range)	n clusters (t*)	% curve (range)	n clusters (t*)	% curve (range)
Spasticity	Hip^a^	2 (2.46)	38% (3–41%)	2 (2.63)	42% (26–68%)	4 (2.75)	6% (0–6%)	-	
			35% (50–85%)		7% (87–94%)		17% (36–53%)		
							11% (65–76%)		
							15% (85–100%)		
	Knee^b^	2 (2.68)	24% (7–31%)	1 (2.94)	40% (28–68%)	4 (3.04)	8% (0–8%)	-	
			27% (55–82%)				27% (30–57%)		
							13% (65–78%)		
							15% (85–100%)		
	Ankle^a^	2 (2.48)	25% (6–31%)	1 (2.67)	5% (88–93%)	3 (2.73)	5% (0–5%)	1 (2.54)	87% (13–100%)
			27% (57–84%)				11% (64–75%)		
							14% (86–100%)		
Weakness	Hip^b^	2 (2.71)	41% (0–41%)	1 (2.89)	65% (32–97%)	4 (2.96)	5% (0–5%)	1 (2.81)	6% (2–8%)
			41% (50–91%)				12% (39–51%)		
							10% (65–75%)		
							16% (84–100%)		
	Knee^b^	2 (2.66)	30% (3–33%)	2 (2.91)	33% (36–69%)	4 (2.96)	6% (0–6%)	-	
			30% (52–82%)		9% (84–93%)		18% (35–53%)		
							12% (64–76%)		
							17% (83–100%)		
	Ankle^b^	2 (2.73)	33% (4–37%)	3 (2.95)	4% (7–11%)	4 (2.97)	6% (0–6%)	-	
			31% (51–82%)		40% (31–71%)		21% (34–55%)		
					11% (82–93%)		11% (65–76%)		
							17% (83–100%)		
Selectivity	Hip^b^	2 (2.72)	39% (0–39%)	1 (2.91)	65% (31–96%)	4 (3.02)	4% (0–4%)	1 (2.92)	5% (1–6%)
			35% (50–85%)				14% (37–51%)		
							5% (68–73%)		
							15% (85–100%)		
	Knee^b^	2 (2.64)	30% (2–32%)	1 (2.91)	68% (28–96%)	4 (3.01)	7% (0–7%)	1 (2.82)	5% (2–7%)
			30% (53–83%)				29% (28–57%)		
							9% (66–75%)		
							17% (83–100%)		
	Ankle^b^	2 (2.75)	33% (3–36%)	2 (2.91)	4% (7–11%)	4 (2.98)	5% (0–5%)	-	
			31% (51–82%)		64% (31–95%)		22% (33–55%)		
							10% (66–76%)		
							17% (83–100%)		
pROM	Hip^a^	2 (2.42)	39% (3–42%)	1 (2.69)	46% (16–62%)	4 (2.72)	8% (0–8%)	-	
			29% (54–83%)				14% (38–52%)		
							12% (64–76%)		
							13% (87–100%)		
	Knee^a^	-		1 (2.65)	41% (20–61%)	2 (2.75)	61% (0–61%)	-	
							4% (96–100%)		
	Ankle^a^	1 (2.42)	13% (24–37%)	1 (2.65)	34% (1–35%)	-		1 (4.05)	100% (0–100%)

pROM: passive range of motion.

^a^ α <0.05

^b^ α < 0.0125

p ≤ 0.05 (orange)

p ≤ 0.01 (blue)

p ≤ 0.001 (green)

n clusters: number of identified suprathreshold clusters; t*: critical threshold needed to reject the null hypothesis; % curve (range): extent of the identified suprathreshold cluster (start and end points of the identified cluster); Hip spasticity: spasticity of the hip flexors; Knee spasticity: spasticity of the knee flexors and extensors; Ankle spasticity: spasticity of the ankle plantarflexors; Hip weakness: weakness of the hip flexors and extensors; Knee weakness: weakness of the knee flexors and extensors; Ankle weakness: weakness of the ankle plantar and dorsiflexors; Hip selectivity: selectivity of the hip flexors and extensors; Knee selectivity: selectivity of the knee flexors and extensors; Ankle selectivity: selectivity of the ankle plantar and dorsiflexors; Hip pROM: pROM of the hip extensors, Knee pROM: knee popliteal angle; Ankle pROM: pROM of the ankle dorsiflexors.

In **uCP**, *pelvic* motion was associated to hip and ankle spasticity (hip: 76%—p < 0.001; ankle: 100%—p <0.001), all joint weakness scores (hip: 89%—p < 0.001; knee: 7%—p = 0.010; ankle: 36%—p = 0.002), knee and ankle selectivity scores (knee: 17%—p = 0.008; ankle: 41%—p = 0.001), and hip and knee pROM scores (hip: 100%—p < 0.001; knee: 5%—p = 0.021) (Table 4, Fig 4, Fig C in S1 File). *Hip* motion showed significant correlations with all joint spasticity scores (hip: 37%—p < 0.001; knee: 18%—p = 0.005; ankle: 15%—p = 0.006 and 21%—p < 0.001), knee and ankle weakness scores (knee: 15%—p = 0.004; ankle: 5%—p = 0.010),knee selectivity (30%—p < 0.001), and all pROM scores (hip: 58%—p < 0.001 and 24%—p = 0.003; knee: 21%—p = 0.003; ankle: 24%—p = 0.003) (Table 4, Fig 5, Fig C in S1 File). *Knee* motion was found to be associated with all joint scores, apart from hip selectivity and all pROM joint scores (Table 4, Fig 6, Fig C in S1 File). *Ankle* motion was related to ankle spasticity (5%—p = 0.007; 84%—p < 0.001), hip and ankle weakness (hip: 15%—p = 0.003; ankle: 3%—p = 0.004 and 18%—p < 0.001), ankle selectivity (3%—p = 0.003 and 19%—p < 0.001), and all pROM joint scores (hip: 28%—p = 0.002 and 29%—p = 0.003; knee: 8%—p = 0.016; ankle: 7%—p = 0.004 and 87%—p < 0.001) (Table 4, Fig 7, Fig C in S1 File). Unique correlations were only identified between knee selectivity and increased *knee* flexion (32–50%), as well as between ankle spasticity and *ankle* motion for a mere 3% of the gait cycle (13–16%).

Children with **bCP** showed, overall, more correlations between joint motions and joint specific impairment scores compared to children with uCP (Table 5, Figs 4–7, Fig D in S1 File). Furthermore, the *pelvis*, *hip* and *knee* motions showed more relationships with the individual joint scores in comparison to the *ankle* motion. In detail, the *pelvic* and *knee* motion were related to all joint scores, apart from the knee and the ankle pROM, respectively, while *hip* motion of children with bCP related to all joint scores. The *ankle* motion was related to ankle spasticity (87%—p < 0.001), hip weakness (6%—p = 0.006), hip and knee selectivity (hip: 5%—p = 0.006; knee: 5%—p = 0.005), and, lastly, ankle pROM (100%—p<0.001). Only two unique correlations were found, namely between increased *hip* flexion with ankle pROM in loading response (1–6%) and increased *knee* flexion with knee pROM during midstance (9–27%).

## Discussion

This study applied an SnPM analysis to identify to what extent specific regions of sagittal plane kinematics during gait are associated to impairments of spasticity, weakness, loss of selectivity and contractures in a large cohort of children with **uCP** and **bCP** (N = 367), across all childhood and with different levels of functional performance (GMFCS levels I-III) and degrees of impairments. The explorations focused on the frequently used measures of spasticity and muscle weakness, as well as on selectivity and pROM; factors which have additionally been shown to have an impact on gait [3,6,9,12,13]. Children with **bCP** were previously found to be more affected by clinical impairments in comparison to **uCP** [10], which is in agreement with current findings. The analyses delineated the relationships separately for uCP and bCP children, highlighting different correlations between impairments and gait between both groups.

### Relation of the composite impairment scores with the combined sagittal motion of the lower limb joints and with each individual joint motion (research question 1)

The analysis of the first aim demonstrated that the *composite scores of spasticity*, *weakness*, *selectivity and pROM*, representing the overall impairments’ severity, were associated with the sagittal motion vector for both **uCP** and **bCP** (Tables 2 and 3, Fig 3, Figs A and B in S1 File). This is in line with findings from a previous study that examined crouch gait in relation to the severity of spasticity, weakness and selectivity [12]. Overall, the sagittal motion of children with **uCP** showed slightly less correlations with the *composite impairment scores* than **bCP** children, which were expressed by smaller and more frequently interrupted suprathreshold clusters, especially for the *composite spasticity*, *weakness and selectivity scores*. Yet, from Fig 3, it becomes clear that the sagittal motion vector is characterized by extensive clusters, covering almost the entire gait cycle, especially in **bCP**. While this finding pointed to the relevance of the overall association between impairments and gait, it lacks specificity to support clinical decision making, and more so with regard to focal treatments. However, the post-hoc analyses revealed correlations of the *composite scores* to each individual joint motion, showing a more detailed picture, and with clear differences between the two groups. These differences may be partly explained by the fact that the *composite impairment scores* were significantly different between them (except for *pROM*).

The post-hoc analyses for the *pelvis* revealed that the *composite spasticity score* in children with **uCP** is related to gait deviations in one large suprathreshold cluster, spanning from midstance till midswing, while the relation for **bCP** spans two separate suprathreshold clusters. This difference most likely reflected the single and double bump patterns, commonly observed in **uCP** and **bCP**, respectively [41–43]. Interestingly, this difference in suprathreshold clusters between the two groups is also observed for the *composite weakness and selectivity scores*, yet, with smaller clusters for **uCP** in comparison to the *composite spasticity score*. Surprisingly, the *pelvis* motion was not found to be associated with the *composite pROM* (except for a relatively short cluster during stance phase in **bCP** children).

The differences between both groups became even more obvious when gradually focusing on the motion of more distal joints. For example, the correlation of *hip* motion to the *composite spasticity score* showed two rather similar suprathreshold clusters for both groups, i.e. one large cluster covering terminal stance and preswing and one short cluster in swing, which could have many reasons (such as a total flexion pattern, a hyperflexion as a reaction of the stretched hip flexors at the end of stance or a compensation strategy for a lack of foot clearance). For the *composite weakness score* of children with **uCP**, the *hip* motion showed only one small cluster during terminal stance, while for the *composite selectivity score* no correlations were found. In contrast, in **bCP** children both *composite weakness and selectivity scores* showed correlations with the *hip* motion from terminal stance to the majority of the swing phase. For the *composite pROM score*, the *hip* motion showed one suprathreshold cluster that is located much later in the gait cycle in **uCP** (cluster covering the transition from stance to swing) than for **bCP** (cluster only covering stance phase).

Clearly different clusters, and also larger differences between uCP and bCP, were observed for the correlations between *knee* and *ankle* motions and the *composite scores*. Detailed study of Fig 3 revealed that the *knee* motion in **uCP** related to the *composite spasticity*, *weakness and selectivity scores* only during the swing phase. In **bCP**, however, additional correlations for these scores were found in stance, especially during loading response and terminal stance. In the same line, in **bCP**, the SnPM post hoc analyses revealed relationships between the *knee* motion and the *composite pROM score*, yet, only for the stance phase of the gait cycle.

The differences in the observed relations between **uCP** and **bCP** are most obvious when focusing on the *ankle* motion. For **uCP**, the *ankle* shows clear clusters in swing phase for all *composite scores*, combined with one additional terminal stance phase cluster for the *composite spasticity score*. For **bCP**, almost no suprathreshold clusters were observed for the *composite scores*.

In summary, the general relationship of the *composite impairment scores* with the combined sagittal motion of the lower limb joints (i.e. the vector) highlights the overall association between lower limb impairments and sagittal gait pathology in CP. The more detailed post-hoc analyses, delineating the relations of the *composite impairment scores* with each individual joint motion, showed suprathreshold clusters that were more obvious at the proximal levels compared to the more distal joints, especially for **uCP**. For **uCP**, the clusters were most obvious during swing phase, while in **bCP**, the clusters were also highly concentrated in stance, especially at terminal stance. A variety of pathological joint motion patterns during stance and swing could potentially be linked to the observed suprathreshold clusters [41]. However, future research is required, including detailed analyses of the observed joint patterns, to fully understand the different relations observed between **uCP** and **bCP**, combining knowledge stemming from similar studies [2,12] and current study results. In general, pROM showed less correlations to joint motions than spasticity, weakness and selectivity, which is most obvious for uCP, where pROM was hardly associated with any of the joint motions, even though the *composite pROM score* was not found to be significantly different between **uCP** and **bCP**.

### Relation of joint motions to their respective joint specific impairment scores and to the impairment scores of other joints (research question 2)

Since previous studies suggested potential associations between the impairments at one joint and motion at another joint [2,3], the second aim of this study was to investigate whether SnPM would also capture this phenomenon in a more systematic way. All joint motions were tested separately for possible relationships with the impairments of the respective joint (here-after labeled as ‘direct’ relations), as well as with the impairments of the neighboring joints (here-after labeled as ‘carry-over’ relations).

Considering the direct relations between *pelvic* motion and the directly involved impairment scores, the image of a single versus a double bump pattern was again observed in the **uCP** and **bCP** groups, respectively. These *pelvis* patterns were directly related to hip spasticity, weakness and pROM for the **uCP** children and to all hip impairment scores for the **bCP** children. Regarding hip flexor spasticity, these single and double bump patterns can occur when hip flexors are stretched as an involved lower limb moves to maximum hip extension (unilateral in **uCP** and bilateral in **bCP**), causing an increase in pelvic anterior tilt (once in **uCP** and twice in **bCP**). The carry-over relations between *pelvis* and the impairment scores of the knee and the ankle were also clearly different among the two groups, indicating different carry-over relationships between *pelvic* motion and more distal impairments. In **uCP**, the carry-over relation was mainly observed for the ankle spasticity score, which was related to *pelvic* motion throughout the entire gait cycle. On the other hand, in **bCP,** stronger carry-over relationships were observed, since the impairments of spasticity, muscle weakness and selectivity of both the knee and ankle were also found to be associated to the “double bump” pattern of the *pelvic* motion.

Regarding *hip* motion, it was expected that the lack of hip extension in terminal stance, which was observed in both groups, would directly associate with hip spasticity and pROM; a hypothesis that was confirmed. For children with **bCP in specifc,** all hip impairment scores directly associated to diminished hip extension in terminal stance, as well as to increased hip flexion during swing. Carry-over relationships of *hip* motion with impairments at the level of the knee and ankle were less prominent in **uCP.** These were limited to knee impairments relating to terminal stance or initial swing *hip* deviations, ankle spasticity relative to loading response and terminal swing hip deviations and ankle pROM relation to swing deviations in the *hip*. On the other hand, in **bCP**, carry-over relationships were quite clear, indicating that deviations at the level of the *hip* were related to impairments at more distal joints.

Similarly, the *knee* motion of the **bCP** children was directly associated with all knee impairments, and with clear suprathreshold clusters at different parts of the gait cycle. Surprisingly, in **uCP**, knee impairments showed limited relationships with *knee* motion, i.e. only in swing for spasticity, weakness and selectivity, and in mid- to terminal stance for selectivity. In **bCP**, several carry-over relations were observed for *knee* motion, including correlations with hip and ankle impairments during loading response, terminal stance and most of swing. In **uCP**, hip and ankle carry-over relationships were relatively small and mainly prominent in the swing phase.

When inspecting the direct relationships between the *ankle* motion and the ankle impairment scores, it became clear that in **uCP,** ankle spasticity and pROM showed the most obvious correlations, with extended suprathreshold clusters covering almost the entire gait cycle. In addition, for weakness and selectivity, direct relations were also observed with small clusters during loading response and terminal swing. In **bCP**, ankle spasticity and pROM were the only two impairments that showed direct relationships with impaired *ankle* motion for almost the entire gait cycle. In **bCP** children, there were hardly any carry-over relationships of the *ankle* motion with the impairment scores of the neighboring joints. These results on carry-over relations were essentially different from the findings at the *pelvis*, *hip* and *knee* motions, indicating that *ankle* deviations were related to intrinsic problems and not greatly influenced by impairments at more proximal levels.

Despite the fact that correlations between muscle scores and gait deviations were not the focus of the present study, it might be interesting to explore whether a focus on specific muscles would further clarify the identified relationships. A few examples of such explorations are reported in Figs E-H in S1 File, where the spasticity of the hamstrings and the rectus femoris muscles were individually explored for their relations with the *knee* sagittal plane kinematics in children with **uCP** (Fig E in S1 File) as well as with **bCP** (Fig F in S1 File). It is shown that only spasticity of the hamstrings correlates with *knee* motion in the **uCP** group. Similarly, the correlations between the weakness scores of the knee flexors and extensors and the *knee* motion was explored in both groups (Figs G-H in S1 File), showing that in children with **uCP**, only the combined weakness of both muscle groups is associated with *knee* deviations and these correlations are not strong enough when the individual muscles are inspected.

In general, the current study results revealed associations between gait and spasticity scores that have been challenging to be mapped in previous studies [2,3,10,11]. For example, spasticity of the plantarflexors has been associated either with a lack of ability for dorsiflexion during stance or with excessive plantarflexion during swing [17,18]. Current study findings illustrated that plantarflexor spasticity was also related to an increased *pelvic* anterior tilt throughout the entire gait cycle in **uCP** and through more than 50% of *pelvic* motion in **bCP**, as well as to increased *hip* flexion and pathological *knee* motion. Associations between ankle spasticity and the motions of the *pelvis* and *knee* joints have also been recently reported for a large cohort of CP children [2], where uCP and bCP were mixed. Our previous study that employed an SPM analysis to compare the gait of CP children (including both **uCP** and **bCP** groups) before and after the administration of botulinum toxin injections to treat spasticity, identified that *knee* flexion during terminal stance and *ankle* dorsiflexion during the majority of the gait cycle improved after spasticity reduction [19]. These results were in line with the results obtained in the current study, where it was shown that the *knee* flexion in the **bCP** group and the *ankle* dorsiflexion in the **uCP** group were associated with the respective *composite spasticity scores* [19]. Moreover, spasticity of the plantarflexors and restricted pROM in both groups correlated with reduced *ankle* dorsiflexion, suggesting a target of botulinum toxin injections in the plantarflexors (and/or combined with casting). In addition, increased *knee* flexion during stance has previously been attributed to spasticity of the hamstrings or the plantarflexors, fixed knee contractures, hip, knee or plantarflexor weakness [17,18] and has also been associated to hip spasticity [2]. All of the above findings were confirmed by the results of this study, except for the knee contractures for the children with **uCP**, even though the knee contractures’ scores were not significantly different between the two groups (Table 1).

Previous research has employed various approaches (e.g. by extracting scalar metrics, such as minimum values or joint angles at specific parts of the gait cycle) to answer similar research questions with quite heterogeneous results [3–5,8]. As mentioned previously, focusing on distinct parameters might increase the likelihood of false positive results [23], but might also not yield any significant results in case of a wrong initial hypothesis. Moreover, incorporating information from the entire gait cycle at once, in the form of an overall quantification measure (i.e. summary indices), might not be precise enough, as there is no information as to which regions of the gait cycle associate more to the clinical impairments. Studies that used the sums of deviations (i.e. composite scores) have mostly focused on associations with functional tests [6,9,21] and not with gait data, as performed in this study. Hence, the current study results offer more detailed information in comparison to previous studies, by providing a comprehensive overview of the most commonly examined primary impairments in clinical practice and their relationships with specific regions of the gait cycle, and separately for children with **uCP** or **bCP**.

### Clinical context

Current results are an aid in appreciating which impairments affect each group as a whole and in which phases during a gait cycle. Individualized treatment planning could use these findings as a guideline and studies focusing on the effects of treatment could further help transfer this knowledge in everyday practice [19]. For example, the overview provided in Figs 3–7 could assist clinicians to decide on their prospective patients’ treatments, based on a variety of factors including but not limited to: a) whether the patients are unilaterally or bilaterally affected, b) the inspected gait deviations through a 3-DGA, c) the assessment of the clinical impairments. The location of the gait deviations during the gait cycle, along with the measurement of the clinical impairments, could assist in clarifying whether a targeted treatment (e.g. administration of botulinum toxin injections to the gastrocnemius muscle, or a targeted strength training for the hip extensors) or a more generalized one (such as selective dorsal rhizotomy or single event multilevel surgery) would be beneficial for a patient.

### Study limitations

Some considerations about the conducted analyses need to be stated. First, several researchers have previously stated that assessing the association between stationary measurement conditions and dynamic assessments (in this case gait), might not be an optimal approach to capture the hypothesized relationships [3,8,44]. This would mean that establishing valid measurements for these impairments during the activity in scope might be needed. However, thus far, there is not sufficient evidence that this could be achieved. Another amelioration in the applied methodology could possibly stem from the application of standardized, instrumented assessments for spasticity or weakness [45,46] or a more validated tool for selectivity [47]. However, these assessments were chosen on the basis that they are the most widely applied in clinical practice, they are easy to obtain and interpret, and have been proven reliable [48,49]. Nevertheless, more quantitative methods to assess impairments should be used to confirm our results and additionally endorse the use of these clinical scales. Further, as the SPM1d software is under development, the linear regression analysis used in this study does not allow the exploration of the combined impact of impairments on gait in the form of a multiple regression analysis. Should this technique be developed, the establishment of the simultaneous effect of impairments on gait in general or specific joint motions could be further facilitated, while providing more elucidating insights on which parameters are mostly responsible for the clinical image. The current study findings should be interpreted with relative caution; several periods of gait deviations during the gait cycle are associated with more than one impairment at a time (Tables 2 – 5, Figs 3 – 7, Figs A-D in S1 File). Since a multiple regression analysis is not yet incorporated in the SPM1d software, it is not possible to deduct whether all correlations would still remain, or some of them would inevitably dominate over others, especially taking the correlations among the impairment scores into consideration (Tables A-C in S2 File). Nevertheless, there are some exceptions that seem to be impairment specific, represented by the unique correlations that were identified.

Moreover, to our knowledge, it is not yet possible to compare the potential identified supra-threshold clusters, apart from referring to their extent (duration) during the gait cycle. Therefore, it was arbitrarily defined that, although statistically significant, clusters that spanned less than 3% of the gait cycle, were not considered as clinically important to make consequential inferences in the quest of improved clinical-decision making. Ideally, a combined score based on the duration of a correlations and the strength of the correlations would be used, which currently is not available. Furthermore, corrections for multiple hypothesis testing were not applied for RQ2, since over-correcting for all the explored hypotheses of RQ2 could lead to an increased likelihood of false negative results [25]. The strength of the current study findings lies in the fact that, even though potentially under-corrected, more than 90% of the identified clusters for both groups had a p value ≤ 0.01; while 43% and 67% of the clusters showed p-values ≤ 0.001 for the **uCP** and **bCP** group, respectively.

The current results indicated that the gait of children with **bCP** seems to be more related to clinical impairments in comparison to **uCP** gait, especially at the proximal level. Understanding which of these impairments or their combinations are mostly contributing to the patients’ compromised gait would be crucial. For feasibility reasons and due to the interdependence between the two lower limbs’ motions, this study focused only on the most affected lower limb of children with **bCP**. One side only has also been used in previous studies exploring associations between impairments and gait in children with CP [2,3,11,14]. Nevertheless, some controversy over the right approach when considering children with bCP has been raised [50]. Future studies might clarify whether the compensations inflicted by the other pathological side have influenced the presented results, by indicating whether single limb associations alter if the interaction between both lower limbs is considered. For uCP, however, it is rather common practice to only focus on the affected lower limb [50], often neglecting the influence of the motion of the unaffected limb on the pathological motion or the accompanying impairments, which might have influenced the current study findings as well. Identifying per each individual patient which factors are contributing to their current deviations would further aid in clinical decision-making and successful treatment planning. This could be achieved by exploring the more in-depth associations between muscle scores and gait deviations, especially with regard to muscle targeted treatment options, such as botulinum toxin injections. Further research should explore several additional relationships before we are truly able to pinpoint which clinical impairments relate to CP gait and how to treat them optimally. Such explorations should focus on including kinetic and electromyographic measurements, which would provide a more thorough understanding as to why each patient walks in a certain way and which muscle groups are used. In addition, the other planes of motion should also be incorporated. Rotational deviations, such as internal hip rotation, widely affect children with CP [51,52] and are often treated based on 3-DGA recommendations [51], necessitating, thus, a better understanding of their underlying relationships with gait itself. These deviations, nevertheless, are more accurate when measured radiographically [53] and, due to the retrospective nature of this study, radiographs were not available for all patients. Finally, CP is caused by a permanent damage to these patients’ brain (before, during or soon after birth). CP gait may result from a combination of reasons including–but not limited to- the sustained brain lesion, the impairments or the patients’ growth [15,37]. Identifying per each individual patient which factors are contributing to their current deviations would further aid in clinical decision making and treatment planning.

## Conclusions

In conclusion, the present study applied a relatively new statistical analysis technique to describe the relationships between measurements of impairment that are typically observed in children with sCP (spasticity, weakness, diminished selectivity and limitations in pROM) and their gait in the sagittal plane. These results proved that all impairment scores (composite or joint scores) associated to the pathological motions of both children with **uCP** and **bCP**, supporting previous research findings, while simultaneously identifying some relationships that have not been detected before. Overall, children with **uCP** showed less correlations with the impairment scores in comparison to **bCP**. All sagittal motion vectors related to large extents to the *composite scores* in question. Further, apart from the *ankle* joint where gait deviations were mainly associated with the respective joint impairments, all other joints showed a lot of carry-over relationships (i.e. relationships between deviations of the investigated joint and the impairment scores of the neighboring joints). This was most obvious in **bCP** and warrants clinical implications. The results thereby supported the added value of using *composite impairment scores* in similar investigations. Future research should investigate whether the simultaneous inspection of these impairments has an added value in the clinical decision-making process, highlighting which impairments or combination thereof is truly associated with gait deviations and to what extent the current findings are a step in the right direction towards individualized treatment planning.

## Supporting information

S1 Checklist STROBE statement–checklist of items that should in included in reports of observational studies. (PDF)

S1 File **Figs A-H depicting the relationships among impairment scores and sagittal plane motion in children with uCP or bCP.** Fig A. Relationship of composite impairment scores with sagittal plane motion in children with uCP. Each row represents another impairment (from top to bottom: composite spasticity score, composite weakness score, composite selectivity score, composite pROM). Column (A) corresponds to the vector field analysis (non-parametric Canonical Correlation analysis); columns B–E correspond to the individual sagittal plane motions of the pelvis, hip, knee and ankle joints, respectively (post-hoc scalar field non-parametric linear regression analyses). For visualization purposes, kinematic data was grouped according to the level of motor impairments, i.e., low impairments (values above percentile 75—green), moderate impairments (values between percentiles 25 and 75—blue), and severe impairments (values below percentile 25—red). The black bars under each kinematic profile indicate the suprathreshold clusters that were formed when the critical threshold (t*) was exceeded and the null hypothesis was, therefore, rejected. Fig B. Relationship of composite impairment scores with sagittal plane motion in children with bCP. Each row represents another impairment (from top to bottom: composite spasticity score, composite weakness score, composite selectivity score, composite pROM). Column (A) corresponds to the vector field analysis (non-parametric Canonical Correlation analysis); columns B–E correspond to the individual sagittal plane motions of the pelvis, hip, knee and ankle joints, respectively (post-hoc scalar field non-parametric linear regression analyses). For visualization purposes, kinematic data was grouped according to the level of motor impairments, i.e., low impairments (values above percentile 75—green), moderate impairments (values between percentiles 25 and 75—blue), and severe impairments (values below percentile 25—red). The black bars under each kinematic profile indicate the suprathreshold clusters that were formed when the critical threshold (t*) was exceeded and the null hypothesis was, therefore, rejected. Fig C. Relationship of each sagittal joint motion with the respective joint’s impairment scores in children with uCP. From left to right: pelvis, hip, knee and ankle motions (columns A-D, respectively), impairments from top to bottom: spasticity, weakness, selectivity, pROM for children with uCP (scalar field non-parametric linear regression analysis). For visualization purposes, kinematic data was grouped according to the level of motor impairments, i.e., low impairments (values above percentile 75—green), moderate impairments (values between percentiles 25 and 75—blue), and severe impairments (values below percentile 25—red). The black bars under each kinematic profile indicate the suprathreshold clusters that were formed when the critical threshold (t*) was exceeded and the null hypothesis was, therefore, rejected.
Fig D. Relationship of each sagittal joint motion with the respective joint’s impairment scores in children with bCP. From left to right: pelvis, hip, knee and ankle motions (columns A-D, respectively), impairments from top to bottom: spasticity, weakness, selectivity, pROM for children with bCP (scalar field non-parametric linear regression analysis). For visualization purposes, kinematic data was grouped according to the level of motor impairments, i.e., low impairments (values above percentile 75—green), moderate impairments (values between percentiles 25 and 75—blue), and severe impairments (values below percentile 25—red). The black bars under each kinematic profile indicate the suprathreshold clusters that were formed when the critical threshold (t*) was exceeded and the null hypothesis was, therefore, rejected. Fig E. Different knee spasticity scores and knee motion for children with uCP. From left to right: knee motion with sum of spasticity scores of hamstrings and rectus femoris muscles, hamstrings’ spasticity score, rectus femoris’ spasticity score (A-C, respectively). For visualization purposes, kinematic data was grouped according to the level of motor impairments, i.e., low impairments (values above percentile 75—green), moderate impairments (values between percentiles 25 and 75—blue), and severe impairments (values below percentile 25—red). The black bars under each kinematic profile indicate the suprathreshold clusters that were formed when the critical threshold (t*) was exceeded and the null hypothesis was, therefore, rejected. Fig F. Different knee spasticity scores and knee motion for children with bCP. From left to right: knee motion with sum of spasticity scores of hamstrings and rectus femoris muscles, hamstrings’ spasticity score, rectus femoris’ spasticity score (A-C, respectively). For visualization purposes, kinematic data was grouped according to the level of motor impairments, i.e., low impairments (values above percentile 75—green), moderate impairments (values between percentiles 25 and 75—blue), and severe impairments (values below percentile 25—red). The black bars under each kinematic profile indicate the suprathreshold clusters that were formed when the critical threshold (t*) was exceeded and the null hypothesis was, therefore, rejected. Fig G. Different knee weakness scores and knee motion for children with uCP. From left to right: knee motion with sum of weakness scores of knee flexors and knee extensors, knee flexors’ weakness score, knee extensors’ weakness score (A-C, respectively). For visualization purposes, kinematic data was grouped according to the level of motor impairments, i.e., low impairments (values above percentile 75—green), moderate impairments (values between percentiles 25 and 75—blue), and severe impairments (values below percentile 25—red). The black bars under each kinematic profile indicate the suprathreshold clusters that were formed when the critical threshold (t*) was exceeded and the null hypothesis was, therefore, rejected. Fig H. Different knee weakness scores and knee motion for children with bCP. From left to right: knee motion with sum of weakness scores of knee flexors and knee extensors, knee flexors’ weakness score, knee extensors’ weakness score (A-C, respectively). For visualization purposes, kinematic data was grouped according to the level of motor impairments, i.e., low impairments (values above percentile 75—green), moderate impairments (values between percentiles 25 and 75—blue), and severe impairments (values below percentile 25—red). The black bars under each kinematic profile indicate the suprathreshold clusters that were formed when the critical threshold (t*) was exceeded and the null hypothesis was, therefore, rejected.
(PDF)

S2 File **Tables A-C depicting the Spearman rank correlations among the impairment scores.**. Table A. Spearman rank correlations identified fair, moderate and very strong correlations among composite impairment scores in children with unilateral (n = 167) and bilateral (n = 200) cerebral palsy. * p ≤ 0.001; Spearman rank correlations identified fair (light grey), moderate(darker grey) and very strong (dark grey) correlations according to [1]; only correlations above 0.30 are displayed. Table B. Spearman rank correlations identified fair and very strong correlations among joint impairment scores in children with unilateral (n = 167) cerebral palsy. * p < 0.001; Spearman rank correlations identified fair (light grey) and very strong (dark grey) correlations according to [1]; only correlations above 0.30 are displayed. Table C. Spearman rank correlations identified fair, moderate and very strong correlations among joint impairment scores in children with bilateral (n = 200) cerebral palsy. * p < 0.001; Spearman rank correlations identified fair (light grey), moderate (darker grey) and very strong (dark grey) correlations according to [1]; only correlations above 0.30 are displayed. [1] Chan YH. Biostatistics 104: Correlational Analysis. SINGAPORE Med J. 2003;44(12):614–9.
(PDF)
