# Supplementary material for: Are spasticity, weakness, selectivity, and passive range of motion related to gait deviations in children with spastic cerebral palsy? A statistical parametric mapping study
Source: PLoS One. 2019 Oct 11;14(10):e0223363. doi: 10.1371/journal.pone.0223363 (PMC6788679; doi:10.1371/journal.pone.0223363)
Supplement: S1 Checklist — (PDF) [file pone.0223363.s001.pdf]

	Item No	Recommendation	Included on page:
**Title and abstract**	1	(*a*) Indicate the study's design with a commonly used term in the title or the abstract	1
		(*b*) Provide in the abstract an informative and balanced summary of what was done and what was found	2
**Introduction**			
Background/rationale	2	Explain the scientific background and rationale for the investigation being reported	3-5
Objectives	3	State specific objectives, including any pre-specified hypotheses	5
**Methods**			
Study design	4	Present key elements of study design early in the paper	6
Setting	5	Describe the setting, locations, and relevant dates, including periods of recruitment, exposure, follow-up, and data collection	6
Participants	6	(*a*) *Cohort study*—Give the eligibility criteria, and the sources and methods of selection of participants. Describe methods of follow-up	6, follow-up not applicable
		Case-control study—Give the eligibility criteria, and the sources and methods of case ascertainment and control selection. Give the rationale for the choice of cases and controls	
		Cross-sectional study—Give the eligibility criteria, and the sources and methods of selection of participants	
		(*b*) *Cohort study*—For matched studies, give matching criteria and number of exposed and unexposed	Not applicable
		Case-control study—For matched studies, give matching criteria and the number of controls per case	
Variables	7	Clearly define all outcomes, exposures, predictors, potential confounders, and effect modifiers. Give diagnostic criteria, if applicable	6-11
Data sources/ measurement	8*	For each variable of interest, give sources of data and details of methods of assessment (measurement). Describe comparability of assessment methods if there is more than one group	6-11
Bias	9	Describe any efforts to address potential sources of bias	Not performed
Study size	10	Explain how the study size was arrived at	6
Quantitative variables	11	Explain how quantitative variables were handled in the analyses. If applicable, describe which groupings were chosen and why	6-11
Statistical methods	12	(*a*) Describe all statistical methods, including those used to control for confounding	10-11
		(*b*) Describe any methods used to examine subgroups and interactions	10-11
		(*c*) Explain how missing data were addressed	Not

			applicable
		(*d*) *Cohort study*—If applicable, explain how loss to follow-up was addressed	Not applicable
		Case-control study—If applicable, explain how matching of cases and controls was addressed	Not applicable
		Cross-sectional study—If applicable, describe analytical methods taking account of sampling strategy	Not applicable
		(*e*) Describe any sensitivity analyses	Not applicable
**Results**			
Participants	13*	(a) Report numbers of individuals at each stage of study—eg numbers potentially eligible, examined for eligibility, confirmed eligible, included in the study, completing follow-up, and analysed	p. 6, Table 1
		(b) Give reasons for non-participation at each stage	Not applicable
		(c) Consider use of a flow diagram	Not relevant
Descriptive data	14*	(a) Give characteristics of study participants (eg demographic, clinical, social) and information on exposures and potential confounders	p. 6, Table 1
		(b) Indicate number of participants with missing data for each variable of interest	Not applicable
		(c) *Cohort study*—Summarise follow-up time (eg, average and total amount)	Not applicable
Outcome data	15*	*Cohort study*—Report numbers of outcome events or summary measures over time	Table 1
		Case-control study—Report numbers in each exposure category, or summary measures of exposure	Not applicable
		Cross-sectional study—Report numbers of outcome events or summary measures	Not applicable
Main results	16	(*a*) Give unadjusted estimates and, if applicable, confounder-adjusted estimates and their precision (eg, 95% confidence interval). Make clear which confounders were adjusted for and why they were included	p. 12-30, including Tables 2-5 and Figs 3-7
		(*b*) Report category boundaries when continuous variables were categorized	Not performed
		(*c*) If relevant, consider translating estimates of relative risk into absolute risk for a meaningful time period	Not applicable
Other analyses	17	Report other analyses done—eg analyses of subgroups and interactions, and sensitivity analyses	p. 12, Table 1
**Discussion**			
Key results	18	Summarise key results with reference to study objectives	30-38
Limitations	19	Discuss limitations of the study, taking into account sources of potential bias or imprecision. Discuss both direction and magnitude of any potential bias	38-40

Interpretation	20	Give a cautious overall interpretation of results considering objectives, limitations, multiplicity of analyses, results from similar studies, and other relevant evidence	40-41
Generalisability	21	Discuss the generalisability (external validity) of the study results	Not performed
**Other information**			
Funding	22	Give the source of funding and the role of the funders for the present study and, if applicable, for the original study on which the present article is based	Submitted online
