# Supplementary figures and images for: Are spasticity, weakness, selectivity, and passive range of motion related to gait deviations in children with spastic cerebral palsy? A statistical parametric mapping study
Source: PLoS One. 2019 Oct 11;14(10):e0223363. doi: 10.1371/journal.pone.0223363 (PMC6788679; doi:10.1371/journal.pone.0223363)

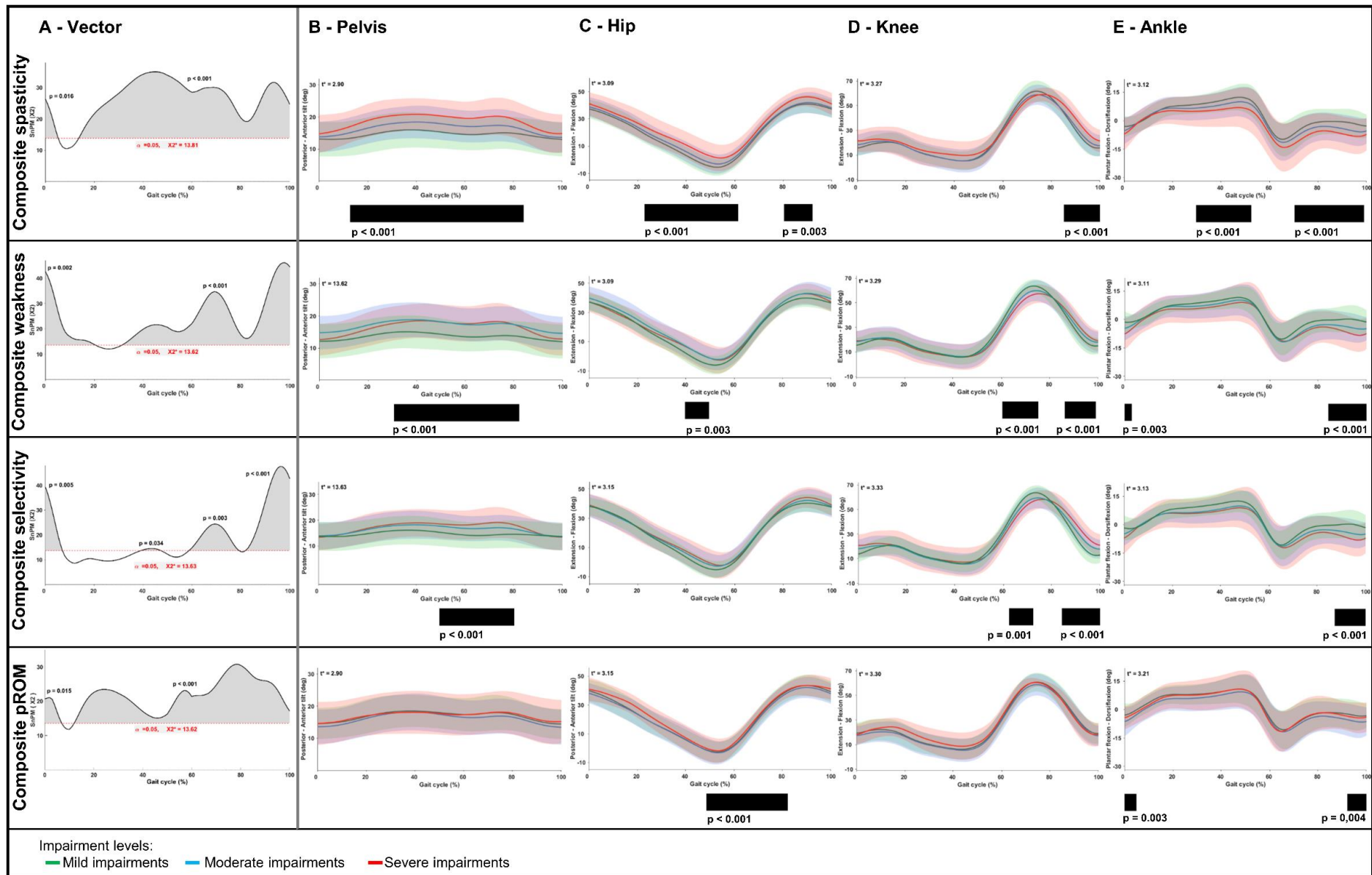

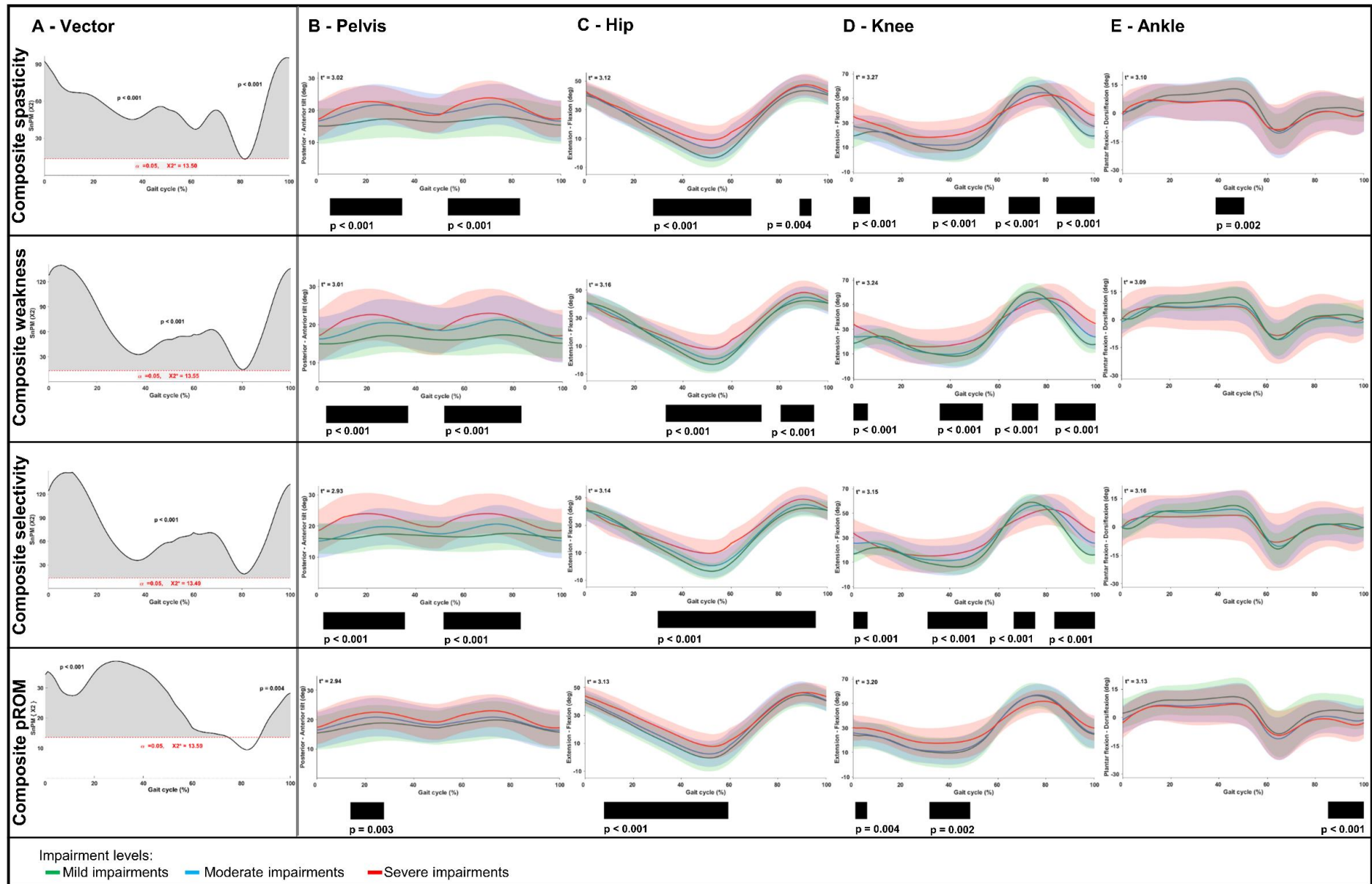

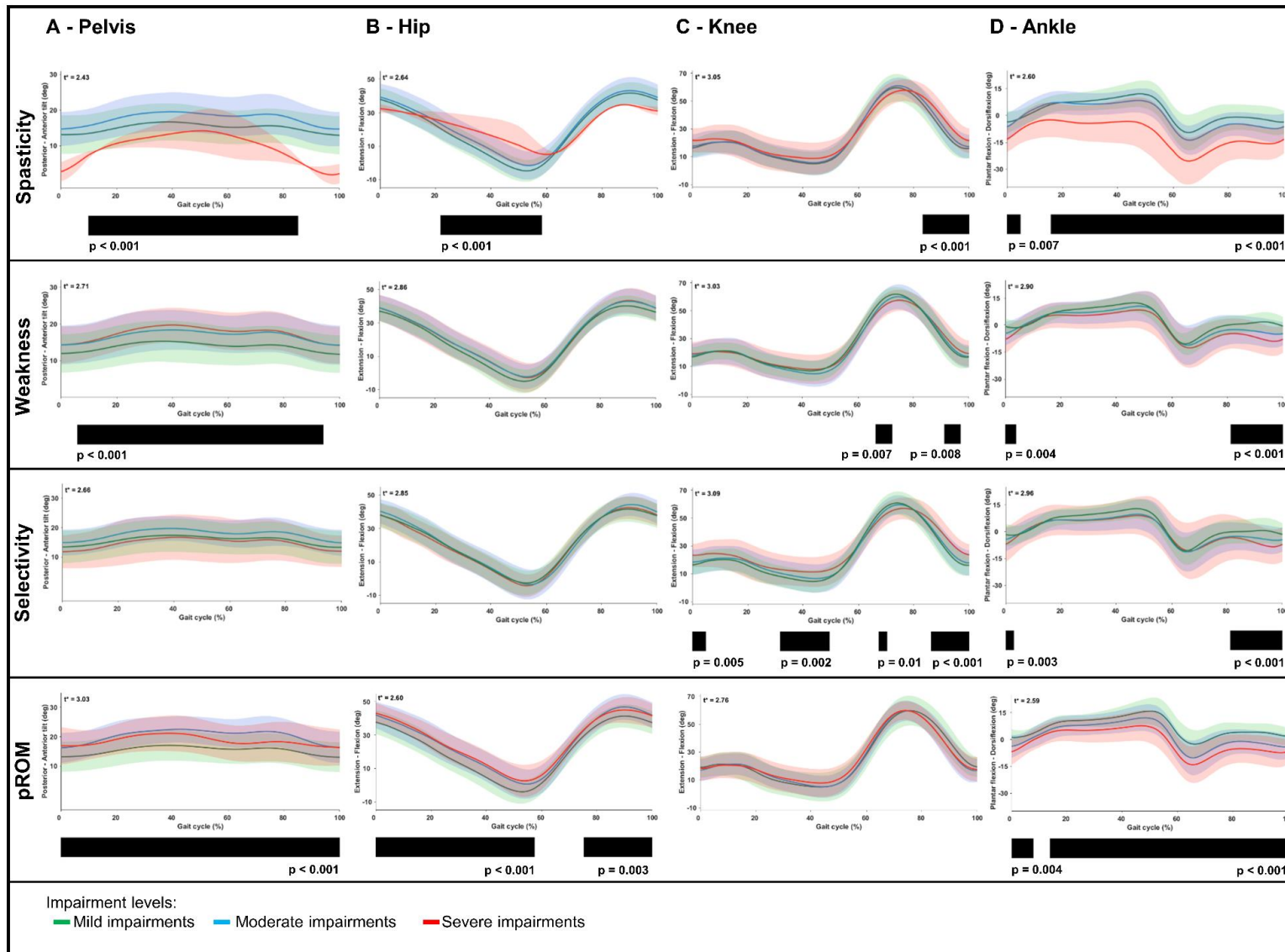

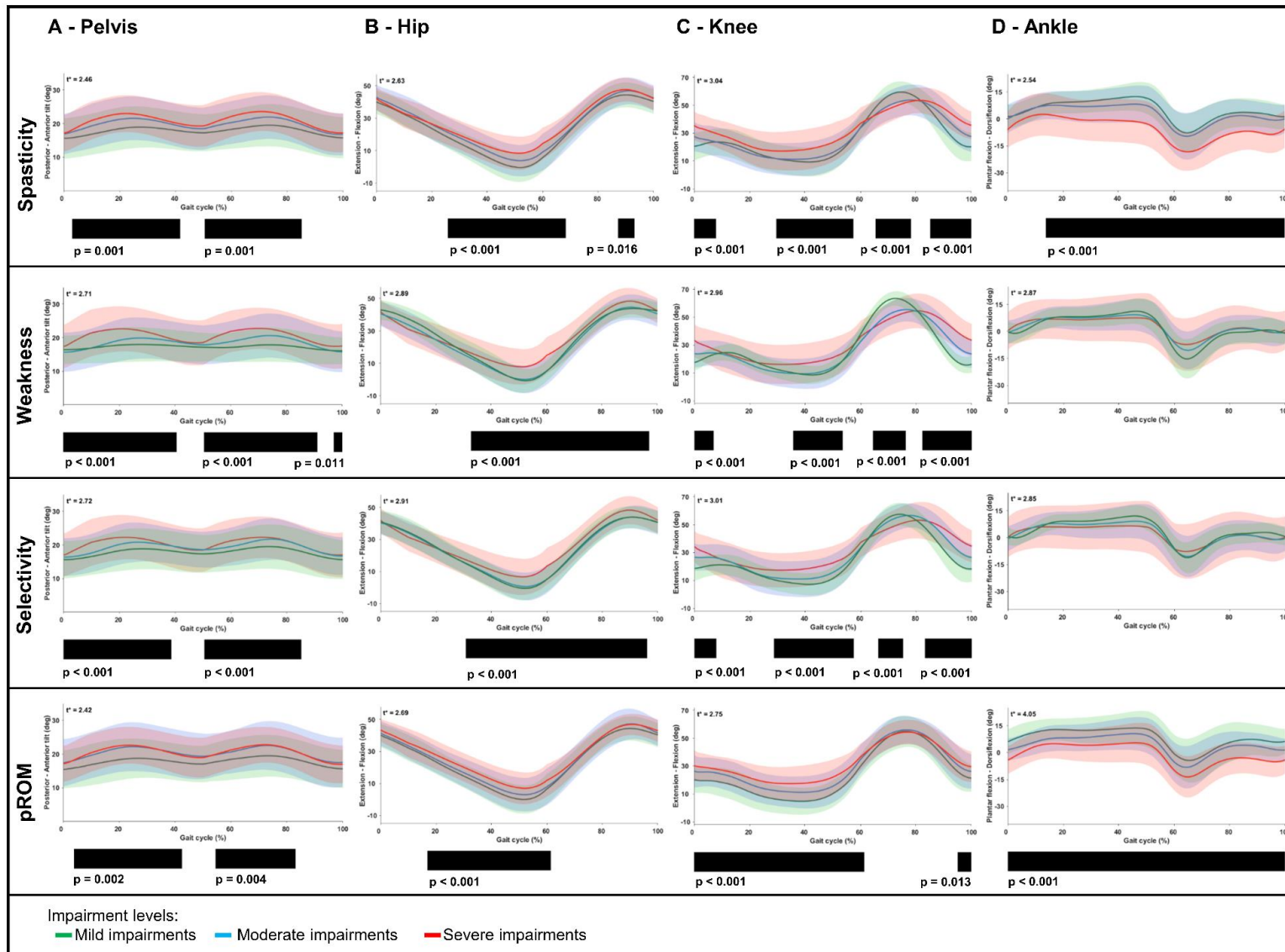

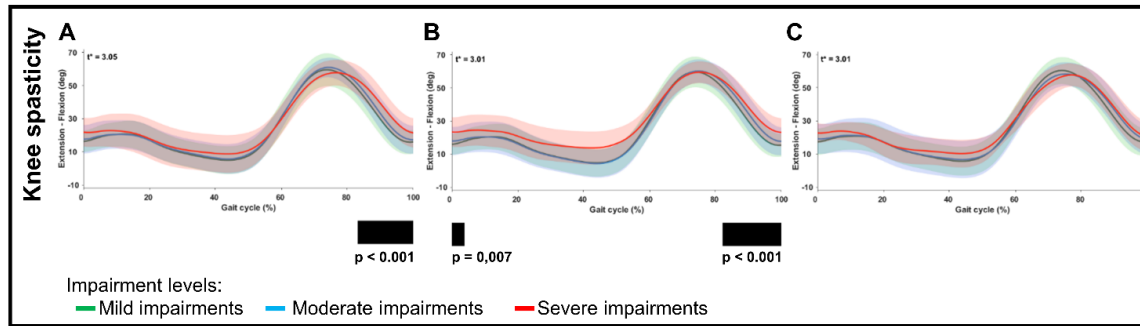

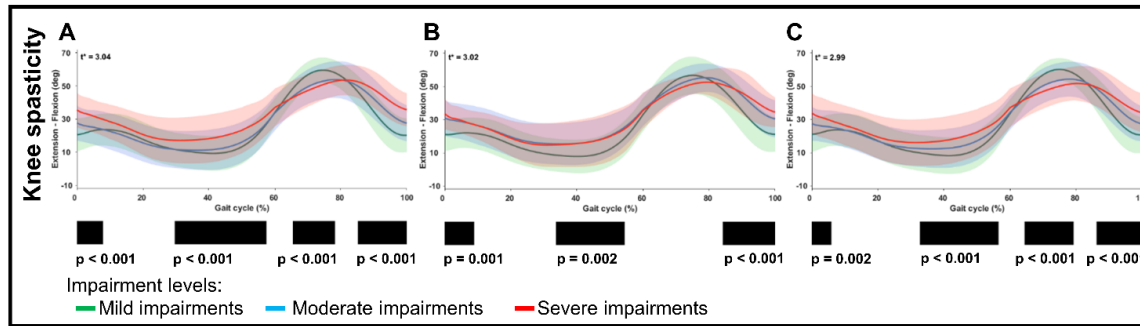

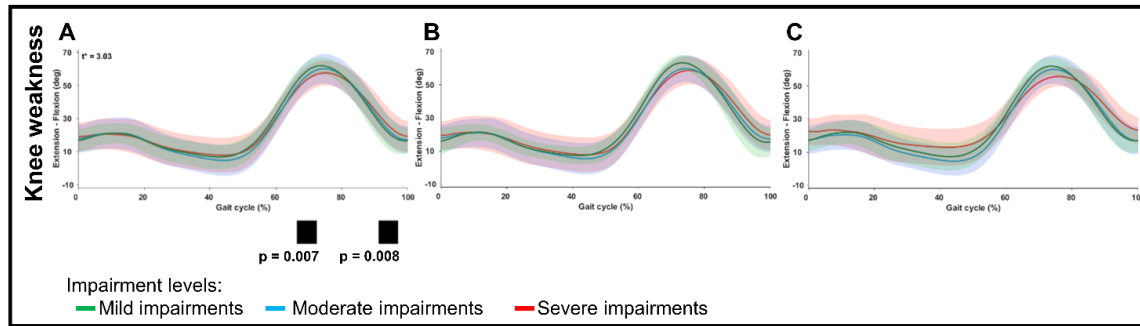

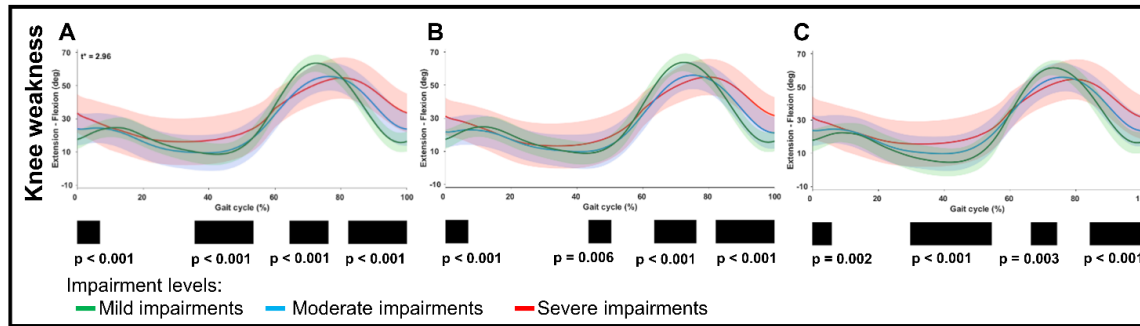

Supplement: S1 File — Figs A-H depicting the relationships among impairment scores and sagittal plane motion in children with uCP or bCP. Fig A. Relationship of composite impairment scores with sagittal plane motion in children with uCP. Each row represents another impairment (from top to bottom: composite spasticity score, composite weakness score, composite selectivity score, composite pROM). Column (A) corresponds to the vector field analysis (non-parametric Canonical Correlation analysis); columns B–E correspond to the individual sagittal plane motions of the pelvis, hip, knee and ankle joints, respectively (post-hoc scalar field non-parametric linear regression analyses). For visualization purposes, kinematic data was grouped according to the level of motor impairments, i.e., low impairments (values above percentile 75—green), moderate impairments (values between percentiles 25 and 75—blue), and severe impairments (values below percentile 25—red). The black bars under each kinematic profile indicate the suprathreshold clusters that were formed when the critical threshold (t*) was exceeded and the null hypothesis was, therefore, rejected. Fig B. Relationship of composite impairment scores with sagittal plane motion in children with bCP. Each row represents another impairment (from top to bottom: composite spasticity score, composite weakness score, composite selectivity score, composite pROM). Column (A) corresponds to the vector field analysis (non-parametric Canonical Correlation analysis); columns B–E correspond to the individual sagittal plane motions of the pelvis, hip, knee and ankle joints, respectively (post-hoc scalar field non-parametric linear regression analyses). For visualization purposes, kinematic data was grouped according to the level of motor impairments, i.e., low impairments (values above percentile 75—green), moderate impairments (values between percentiles 25 and 75—blue), and severe impairments (values below percentile 25—red). The black bars under each kinema [file pone.0223363.s002.pdf]
