# Supplementary material for: Are spasticity, weakness, selectivity, and passive range of motion related to gait deviations in children with spastic cerebral palsy? A statistical parametric mapping study
Source: PLoS One. 2019 Oct 11;14(10):e0223363. doi: 10.1371/journal.pone.0223363 (PMC6788679; doi:10.1371/journal.pone.0223363)
Supplement: S2 File — Tables A-C depicting the Spearman rank correlations among the impairment scores.. Table A. Spearman rank correlations identified fair, moderate and very strong correlations among composite impairment scores in children with unilateral (n = 167) and bilateral (n = 200) cerebral palsy. * p ≤ 0.001; Spearman rank correlations identified fair (light grey), moderate(darker grey) and very strong (dark grey) correlations according to [1]; only correlations above 0.30 are displayed. Table B. Spearman rank correlations identified fair and very strong correlations among joint impairment scores in children with unilateral (n = 167) cerebral palsy. * p < 0.001; Spearman rank correlations identified fair (light grey) and very strong (dark grey) correlations according to [1]; only correlations above 0.30 are displayed. Table C. Spearman rank correlations identified fair, moderate and very strong correlations among joint impairment scores in children with bilateral (n = 200) cerebral palsy. * p < 0.001; Spearman rank correlations identified fair (light grey), moderate (darker grey) and very strong (dark grey) correlations according to [1]; only correlations above 0.30 are displayed. [1] Chan YH. Biostatistics 104: Correlational Analysis. SINGAPORE Med J. 2003;44(12):614–9. (PDF) [file pone.0223363.s003.pdf]

		Composite spasticity	Composite weakness	Composite selectivity	Composite pROM
uCP	Composite spasticity				
	Composite weakness	-0.39*			
	Composite selectivity	-0.41*	0.83*		
	Composite pROM	0.46*			
bCP	Composite spasticity				
	Composite weakness	-0.57*			
	Composite selectivity	-0.60*	0.90*		
	Composite pROM	0.53*			

	Joint spasticity scores			Joint weakness scores			Joint selectivity scores			Joint pROM scores		
	Hip	Knee	Ankle	Hip	Knee	Ankle	Hip	Knee	Ankle	Hip	Knee	Ankle
Joint spasticity scores												
Hip												
Knee	0.31*											
Ankle												
Joint weakness scores												
Hip												
Knee	0.32*			0.50*								
Ankle		0.35*		0.39*	0.53*							
Joint selectivity scores												
Hip				0.51*		0.36*						
Knee	0.36*	0.32*		0.30*	0.53*	0.51*	0.33*					
Ankle				0.34*	0.47*	0.90*	0.38*	0.48*				
Joint pROM scores												
Hip	0.52*							0.30*				
Knee		0.37*										
Ankle											0.30*	

	Joint spasticity scores			Joint weakness scores			Joint selectivity scores			Joint pROM scores		
	Hip	Knee	Ankle	Hip	Knee	Ankle	Hip	Knee	Ankle	Hip	Knee	Ankle
Joint spasticity scores												
Hip												
Knee	0.54*											
Ankle	0.32*	0.44*										
Joint weakness scores												
Hip	0.34*	0.46*										
Knee	0.39*	0.52*		0.76*								
Ankle	0.36*	0.54*		0.73*	0.75*							
Joint selectivity scores												
Hip	0.34*	0.45*		0.76*	0.72*	0.66*						
Knee	0.43*	0.58*	-0.30*	0.67*	0.77*	0.70*	0.76*					
Ankle	0.34*	0.56*	-0.36*	0.69*	0.71*	0.90*	0.69*	0.74*				
Joint pROM scores												
Hip	0.68*	0.35*						-0.31*				
Knee	0.30*	0.44*										
Ankle												
